# Generation and Functional *In Vitro* Analysis of Semliki Forest Virus Vectors Encoding TNF-α and IFN-γ

**DOI:** 10.3389/fimmu.2017.01667

**Published:** 2017-11-30

**Authors:** Baiba Kurena, Elisabeth Müller, Panagiotis F. Christopoulos, Ingvild Bjellmo Johnsen, Branislava Stankovic, Inger Øynebråten, Alexandre Corthay, Anna Zajakina

**Affiliations:** ^1^Tumor Immunology Lab, Department of Pathology, Rikshospitalet, Oslo University Hospital, University of Oslo, Oslo, Norway; ^2^Cancer Gene Therapy Group, Latvian Biomedical Research and Study Centre, Riga, Latvia; ^3^Department of Biosciences, University of Oslo, Oslo, Norway; ^4^Department of Laboratory Medicine, Norwegian University of Science and Technology, Trondheim, Norway

**Keywords:** Semliki Forest virus, cancer immunotherapy, gene delivery, cytokines, macrophage activation, Lewis lung carcinoma

## Abstract

Cytokine gene delivery by viral vectors is a promising novel strategy for cancer immunotherapy. Semliki Forest virus (SFV) has many advantages as a delivery vector, including the ability to (i) induce p53-independent killing of tumor cells *via* apoptosis, (ii) elicit a type-I interferon (IFN) response, and (iii) express high levels of the transgene. SFV vectors encoding cytokines such as interleukin (IL)-12 have shown promising therapeutic responses in experimental tumor models. Here, we developed two new recombinant SFV vectors encoding either murine tumor necrosis factor-α (TNF-α) or murine interferon-γ (IFN-γ), two cytokines with documented immunostimulatory and antitumor activity. The SFV vector showed high infection rate and cytotoxicity in mouse and human lung carcinoma cells *in vitro*. By contrast, mouse and human macrophages were resistant to infection with SFV. The recombinant SFV vectors directly inhibited mouse lung carcinoma cell growth *in vitro*, while exploiting the cancer cells for production of SFV vector-encoded cytokines. The functionality of SFV vector-derived TNF-α was confirmed through successful induction of cell death in TNF-α-sensitive fibroblasts in a concentration-dependent manner. SFV vector-derived IFN-γ activated macrophages toward a tumoricidal phenotype leading to suppressed Lewis lung carcinoma cell growth *in vitro* in a concentration-dependent manner. The ability of SFV to provide functional cytokines and infect tumor cells but not macrophages suggests that SFV may be very useful for cancer immunotherapy employing tumor-infiltrating macrophages.

## Introduction

Viral vectors have been used for cancer immunotherapy to intratumorally deliver and express transgenes and to thereby increase therapeutic protein delivery while simultaneously reducing systemic toxicity (1). Recombinant Semliki Forest virus (rSFV) has been successfully used as a cancer immunotherapy in preclinical tumor models for cytokine gene delivery (2–15) and for immunization with tumor-associated antigens against mastocytoma (2, 16) and human papilloma virus (HPV)-associated cervical cancer (17, 18). Semliki Forest virus (SFV) belongs to the *Alphavirus* genus of the *Togaviridae* family and possesses an enveloped nucleocapsid that contains a positive-sense single-stranded (+ss) RNA genome (19). The replication-deficient SFV vector system delivers genes of interest by infecting the cells with viral particles and thereby providing a transiently high level of transgene expression without further virus replication (20). The SFV-based vector is an attractive tool for cancer immunotherapy because of its oncolytic nature and ability to induce *p53*-independent apoptosis of tumor cells (21), which may facilitate uptake and presentation of tumor antigens from the apoptotic bodies by antigen-presenting cells, thus enhancing cancer immunogenicity (22, 23). In addition, SFV vector elicits endogenous type-I interferon (IFN) responses, which may be required for the therapeutic effect of a vector-encoded cytokine (3).

Cytokines can contribute to antitumor immune responses in the tumor microenvironment (24, 25) and are, therefore, promising tools for cancer immunotherapy. rSFV has been used in preclinical studies to modulate the tumor microenvironment by locally delivering cytokines, such as interleukin (IL)-12 (2–11), IL-18 (12), IFN-α (13), granulocyte-macrophage colony-stimulating factor (14), and endostatin (15). An SFV vector encoding the heterodimeric murine IL-12 (SFV-IL12) was shown to have a strong therapeutic antitumor effect when locally injected in tumor models of melanoma (4), glioma (5), colon adenocarcinoma (3, 6, 7), hepatic colon adenocarcinoma (8), hepatocellular carcinoma (9), mastocytoma (2), fibrosarcoma (7), breast carcinoma (7), and lung carcinoma (3) in mice, orthotopic hepatocellular carcinoma in rats (10), and spontaneous hepatocellular carcinoma in woodchucks (11). The therapeutic activity of SFV-IL12 involved immune responses that were mediated by IL-12 in combination with the virus-induced endogenous type-I IFN (3, 9, 11), which lead to an angiostatic effect (4) and enhanced CD8+ T-cell responses (3, 5–8, 11).

Two well-known cytokines that may be potentially useful for cancer immunotherapy are IFN-γ and tumor necrosis factor-α (TNF-α). The pleiotropic cytokine IFN-γ is a tumor-suppressive factor that protects against carcinogen-induced and spontaneous tumor development (26, 27), inhibits tumor angiogenesis (28–31) and may improve tumor immunogenicity by upregulating major histocompatibility complex class I molecules (32, 33). Furthermore, it has been shown that IFN-γ enhances macrophage activation toward a tumoricidal phenotype *in vitro* (34–36) and *in vivo* (31, 37–39). TNF-α was discovered in 1975 as a serum factor inducing haemorrhagic necrosis in tumors (40) and, therefore, this cytokine was proposed as a potential anti-cancer agent. TNF-α has been shown not only to selectively destroy tumor vasculature (41, 42), but also increase tumor vessel permeability, thus, improving drug penetration into tumors (43–45). Moreover, low doses of TNF-α have been shown to promote antitumor immune responses by enhancing T-cell infiltration and by activating macrophages toward a tumor-suppressive phenotype (46). Notably, a synergistic action of IFN-γ and TNF-α was reported in early studies showing tumor growth inhibition in mice (47, 48) and tumor disappearance in patients after local limb perfusions with IFN-γ and TNF-α in combination with a chemotherapeutic agent (49). The antitumor effects were likely due to decreased endothelial cell adhesion and survival in response to TNF-α and IFN-γ leading to destruction of tumor vasculature (50). The synergism may also be explained by the fact that IFN-γ enhances TNF-α receptor expression in malignant cells (51, 52), thus improving TNF-α treatment. Another synergistic action of IFN-γ and TNF-α has been shown on macrophage activation toward a tumoricidal phenotype *in vitro* (53). However, the clinical usefulness of TNF-α and IFN-γ is limited by their systemic toxicity (54, 55) and short *in vivo* half-lives (56, 57).

To the best of our knowledge, no previous studies have reported using rSFV vectors that encode the cytokine IFN-γ or TNF-α. To provide new tools for cancer immunotherapy, we developed two rSFV vectors that encoded either murine TNF-α or IFN-γ and tested the functionality of the resulting rSFV-encoded cytokines *in vitro*. Here, we show that the rSFV vectors were capable of infecting Lewis lung carcinoma (LLC) cells and exploiting them to produce rSFV-encoded TNF-α and IFN-γ. Moreover, in this study, we show that rSFV-encoded IFN-γ effectively enhanced macrophage activation toward a cancer-suppressive phenotype *in vitro*.

## Materials and Methods

### Cell Lines and Cell Cultures

Baby hamster kidney (BHK-21) fibroblasts were obtained from American Type Culture Collection (Cat. No. CCL-10™; ATCC/LGC Prochem, Boras, Sweden) and cultured in Glasgow’s MEM (Cat. No. 11710; Thermo Fisher Scientific, Boston, MA, USA) supplemented with 10% fetal bovine serum (FBS; Cat. No. F7524; Sigma-Aldrich, St. Louis, MO, USA), 10% Tryptose Phosphate Broth solution (Cat. No. T4049; Sigma-Aldrich), 20 mM HEPES (Cat. No. H0887; Sigma-Aldrich), 2 mM l-Glutamine (Cat. No. 25030; Thermo Fisher Scientific), 100 U/mL penicillin, and 100 mg/mL streptomycin (Cat. No. P0781, Sigma-Aldrich). The LLC cell line (also called LLC1; Cat. No. CLS 400263) and mouse macrophage cell line J774A.1 (Cat. No. CLS 400220) were obtained from Cell Lines Service (CLS GmbH, Eppelheim, Germany). The LLC cells were grown in RPMI-1640 medium (Cat. No. 61870; Thermo Fisher Scientific) supplemented with 10% FBS (Sigma-Aldrich). The J774A.1 cells were cultured in RPMI-1640 medium supplemented with 10% FBS (Cat. No. S0415; Biochrom GmbH, Berlin, Germany). The human lung carcinoma cell line A549 (Cat. No. CCL-185™) and murine fibrosarcoma cell line L929 (Cat. No. CCL-1™) were obtained from American Type Culture Collection (ATCC/LGC Standards GmbH, Wesel, Germany). The A549 cells were propagated in Advanced Dulbecco’s MEM (Cat. No. 12491; Thermo Fisher Scientific) supplemented with 5% FBS (Sigma-Aldrich) and 2 mM L-glutamine. The conditioned medium (CM) of L929 cells was used as a source of macrophage colony-stimulating factor (M-CSF) (58) for the maintenance and differentiation of bone marrow-derived progenitors into macrophages. Briefly, L929-CM was produced by cultivating L929 cells in RPMI-1640 with 10% FBS (Sigma-Aldrich), 100 U/mL penicillin and 100 mg/mL streptomycin in T75 flasks until confluency. The medium was then removed, 30 mL of RPMI-1640 supplemented with 10% FBS (Biochrom) without antibiotics was added, and cells were incubated for 10 days. The L929-CM was harvested and spun at 300 *g* for 10 min. The collected supernatant was filtered through a 0.22-µm strainer and stored at −20°C until used. All cells were cultured at 37°C in a humidified incubator in an atmosphere containing 5% CO_2_ and 95% air.

### Mice

C57BL/6NRj mice (Janvier Labs, Le Genest-Saint-Isle, France) were bred at the Department of Comparative Medicine, Oslo University Hospital, Rikshospitalet (Oslo, Norway). All animal experiments were approved by and performed in accordance with the regulations and guidelines of the Norwegian Food Safety Authority.

### Isolation and Culturing of Bone Marrow-Derived Macrophages (BMDMs)

Murine BMDMs were differentiated from bone marrow progenitors obtained from C57BL/6NRj mice as previously described (59, 60) with a few modifications. Femur and tibia were aseptically dissected from 8- to 10-week-old C57BL/6NRj mice, and bone marrow cells were collected by flushing the femurs and tibias with RPMI-1640 supplemented with 10% FBS (Biochrom) using a 25 G needle. After the cells were centrifuged for 5 min at 400 *g*, the erythrocytes were lysed in lysis buffer (150 mM NH_4_Cl, 10 mM KHCO_3_, and 0.1 mM EDTA-Na_2_, pH 7.2–7.4, filtered through a 0.2-µm strainer), and the remaining cells were then filtered through a 70-µm cell strainer (Cat. No. CLS431751; Sigma-Aldrich) and centrifuged. The cells were seeded in 90-mm untreated cell culture dishes (Cat. No. 734-2359; VWR, Radnor, PA, USA) at a concentration of 8 × 10^6^/dish and then differentiated *via* cultivation for 7 days in medium referred to hereafter as complete BMDM differentiation medium (consisting of RPMI-1640 with 10% FBS and 30% L929-CM containing M-CSF). The adherent cells were considered CD11b+F4/80+ macrophages since flow cytometry revealed that these cells were more than 99% pure (data not shown). After 7 days, the cells were detached by incubating them in cold Dulbecco’s phosphate-buffered saline without Mg^2+^/Ca^2+^ (referred to as PBS−/−; Cat. No. D8537; Sigma-Aldrich) for 15–20 min at 4°C. The harvested cells were centrifuged and frozen in FBS containing 10% DMSO (Cat. No. 0231; VWR). The BMDMs were cultivated in RPMI-1640 supplemented with 10% FBS and 10% L929-CM.

### Generation of Human Monocyte-Derived Macrophages (HMDMs)

Peripheral blood mononuclear cells (PBMCs) were isolated from human buffy coats *via* centrifugation in Lymphoprep™ density gradient medium (Cat. No. 1114547; Alere Technologies AS, Oslo, Norway) according to the manufacturer’s protocol. Buffy coats were obtained from the blood bank of St. Olav’s Hospital (Trondheim, Norway). Monocytes were enriched from total PBMCs *via* plastic adherence in 8-well TC Lab-Tek Chamberslides (Cat. No. 80826; ibidi GmbH, Martinsried, Germany) and maintained in RPMI 1640 medium supplemented with 10% human serum (obtained from the blood bank of St. Olav’s Hospital, Trondheim, Norway). Monocyte-derived macrophages were differentiated from monocytes by incubating the cells in RPMI 1640 containing 10% human serum and 10 ng/mL human M-CSF (Cat. No. SRP6165; Sigma-Aldrich) for 7 days.

### Plasmids and Construction of Expression Vectors

The pSFV1-DsRed vector, which carries a gene for *Discosoma* sp. red fluorescent protein (*DsRed*), was generated in our lab as previously described (61) using the pSFV1 vector (20). The pSFV1 and pSFV-Helper1 plasmids (20) were generously provided by Garoff (Karolinska Institute, Stockholm, Sweden).

To generate the pSFV1-Tnfa-Flag plasmid, we used the pSFV1-NruI vector, which is a derivate of pSFV1 that was generated in our lab by deleting a 527 bp *Stu*I-*Hind*III fragment from pSFV1 (bp 7603–8130) and changing a unique *Spe*I site to a *Nru*I site (62). The murine TNF-α-coding gene (*Tnfa*) (GenBank No. NM_013693.2) fused to a Flag-tag sequence was inserted into the pSFV1-NruI vector under the control of the SFV 26S subgenomic promoter. The *Tnfa-Flag* fragment was amplified from the template plasmid pCMV3-mTNF-Flag (Cat. No. MG50349-CF; Sino Biological Inc., Beijing, China) using PCR with the following primers (synthesized by Microsynth AG, Balgach, Switzerland): 5′-GC**GGATCC**ATGAGCACAGAAAGCATGATC-3′ (forward) and 5′-TG**CCCGGG**TTTACTTATCGTCGTCATCCTTG-3′ (reverse). *BamH*I and *Sma*I restriction sites (shown here in bold) were introduced to facilitate the ligation of the PCR fragment into the pSFV1-NruI vector.

The pSFV1/Enh-Luc plasmid was generously provided by A. Merits (Institute of Technology, University of Tartu, Estonia) and has been previously described (63). The pSFV1/Enh-Ifng vector was generated by replacing the *Xma*I-*Apa*I fragment (bp 7572-9268), which contained the firefly luciferase gene (*Luc*) of pSFV1/Enh-Luc, with the murine IFN-γ-coding gene (*Ifng*) (GenBank No. NM_008337.3). The *Ifng* gene was amplified from the template plasmid pMD19-mIFNG (Cat. No. MG50709-M; Sino Biological Inc.) using PCR with the following primers (synthesized by Microsynth AG): 5′-TCCG**CCCGGG**ATGAACGCTACACACTGC-3′ (forward) and 5′-TCC**GGGCCC**TCAGCAGCGACTCCTTTTCC-3′ (reverse). *Xma*I and *Apa*I (shown here in bold) restriction sites were incorporated to facilitate cloning into the pSFV1/Enh-Luc vector. The sequences *Tnfa-Flag* and *Enh-2A-Ifng* of the constructs were verified by GATC Biotech AG (Constance, Germany) and found to share 100% identity with the reference sequences *Tnfa* (GenBank No. NM_013693.2), *Flag* (shown in the product description of pCMV3-mTNF-Flag (Cat. No. MG50349-CF; Sino Biological)), *Enh-2A* [shown in Ref. (7)] and *Ifng* (NM_008337.3).

### RNA Synthesis and Production of Viral Particles

RNAs were synthesized and transfected into BHK-21 cells using electroporation as previously described (63). The plasmids pSFV/Enh-Ifng, pSFV-DsRed, and pSFV-Helper1 were linearized using the restriction enzyme *SpeI* (Cat. No. ER1251; Thermo Fisher Scientific), and pSFV/Tnfa-Flag was linearized using *NruI* (Cat. No. ER0111; Thermo Fisher Scientific). The linearized plasmids (1 µg of each) were used as templates for the *in vitro* transcription (SP6 RNA polymerase; Cat. No. AM2071; Thermo Fisher Scientific) of SFV-Helper1 RNA and recombinant RNAs (rRNAs) carrying *Ifng, Tnfa*, or *DsRed*. The transcribed rRNAs were capped by adding 1 mM 3′-*O*-Me-m^7^G(5′)ppp(5′)G cap-structure analog (Cat. No. S1411S; New England Biolabs, Hitchin, UK) during the transcription reaction. To package the rRNAs into viral particles, each of the rRNAs was co-electroporated with the SFV-Helper1 RNA in 1 × 10^7^ BHK-21 cells by pulsing the mixture twice at 850 V, 25 mF using a Gene Pulser-II apparatus (Bio-Rad, Hercules, CA, USA). The Helper1 RNA provided *in trans* the SFV structural proteins (20) for encapsidation of the rRNA. The electroporated BHK-21 cells were resuspended in 15 mL of BHK-21 cultivation medium containing 1% FBS (Sigma-Aldrich) and then incubated at 33°C for 48 h. The cell growth medium containing the infectious rSFV particles was then harvested, rapidly frozen in liquid nitrogen, and subsequently used as a source of rSFV particles. The titers of the rSFV particles were determined based on the “one virus particle—one infected cell” hypothesis regarding the replication-deficient virus particles. BHK-21 cells were infected with serial dilutions of the rSFV particles. Infected cells expressing DsRed were counted in 10 viewfields using fluorescence microscopy at 24 h post-infection. The cells that were infected with either SFV-Tnfa or SFV-Ifng were stained with rabbit polyclonal antibodies that were specific for the nsp1 subunit of SFV replicase (generously provided by A. Merits, Institute of Technology, University of Tartu, Estonia) and with a fluorochrome-conjugated secondary antibody (Cat. No. A11034; Thermo Fisher Scientific). The stained cell monolayers were observed using EVOSfl digital inverted fluorescence microscope (Thermo Fisher Scientific), and infection-positive cells were counted in 10 viewfields using fluorescence microscopy. The rSFV particle titers were expressed as infectious units (IFU)/mL relative to virus infectivity in BHK-21 cells and were used later in this study to calculate multiplicity of infection (MOI), which indicated how many infectious viral particles were added per target cell during infection. Cells infected with the rSFV stocks did not secrete infectious virus particles, as shown in cell reinfection experiments, confirming that the virus stocks contained replication-deficient SFV.

### Cancer Cell and Macrophage Susceptibility to SFV and Cell Viability after Infection

Baby hamster kidney, LLC, A549, and J774A.1 cells and murine BMDMs were seeded in 12-well plates (Cat. No. 150628; Thermo Fisher Scientific) or 24-well plates (Cat. No. 142475; Thermo Fisher Scientific) and cultivated until 50–80% confluency. The cells were washed once with PBS containing Mg^2+^ and Ca^2+^ (referred to as PBS+/+; Cat. No. 14040; Thermo Fisher Scientific), and 140—600 µL of an infectious suspension containing SFV-DsRed at a MOI = 10 or MOI = 15 (defined as 10 or 15 IFU in BHK-21 cells) in PBS+/+ was added to duplicate sets of wells. PBS+/+ was used as a negative control. After the cells were incubated at 37°C for 80 min, the infectious suspension was replaced with cell-specific growth medium, and the expression of DsRed in the infected cells was observed 24–48 h later using an EVOSfl digital inverted fluorescence microscope (AMG). The LLC and J774A.1 cells were detached *via* scraping, the BHK-21 cells were detached using a 0.25% trypsin-EDTA solution (Cat. No. T4049; Sigma-Aldrich), and the BMDMs and A549 cells were detached by treatment with accutase (Cat. No. A6964; Sigma-Aldrich). The cells were then centrifuged and DsRed-positive cells were quantified 24 h post-infection using BD FACSCalibur flow cytometer (BD Biosciences, San Jose, CA, USA). To evaluate cell viability BHK-21 and LLC cells were labeled 48 h post-infection with 1 µg/mL of fluorescein isothiocyanate (FITC)-conjugated annexin V (annexin V-FITC from Apoptosis Detection Kit; Cat. No. 556547; BD Biosciences) for 15 min followed by staining with 3 µM of DAPI (Cat. No. 422801; BioLegend, San Diego, CA, USA) for 5 min. Cells were immediately quantified using BD LSRFortessa flow cytometer (BD Biosciences). The data were analyzed using FlowJo V10 software (FlowJo LLC., Ashland, OR, USA).

After differentiation in 8-well TC Lab-Tek Chamberslides (Cat. No. 80826; ibidi GmbH), HMDMs were washed once with PBS+/+ and incubated for 1 h with 200 µL of an infectious suspension containing SFV-DsRed at a MOI = 15 (defined as 15 IFU in BHK-21 cells) diluted in PBS+/+. A recombinant paramyxoviral vector Sendai virus (SeV)-GFP that was based on a wild-type infectious SeV clone that carried a supplemental GFP transgene (64) was generously provided by D. Kolakofsky (Department of Microbiology and Molecular Medicine, University of Geneva School of Medicine, Geneva, Switzerland) and used at a MOI = 15 as a positive control for HMDM infection. The infectious suspension was replaced with normal cell growth medium, and the cells were observed at 24 h post-infection using phase-contrast and confocal fluorescence microscopy with a Zeiss Axiovert 100-M inverted microscope equipped with a LSM 510 laser-scanning unit and a 1.4 NA × 63 Plan-Apochromat oil-immersion objective. To calculate the percentage of fluorescent protein-expressing cells, the total number of cells was manually counted in at least 10 viewfields using phase-contrast microscopy. Fluorescent protein-expressing cells were simultaneously counted within the same viewfields using fluorescence microscopy.

### Cancer Cell Growth Inhibition after Infection with SFV Vectors

Baby hamster kidney cells (1.97 × 10^4^ cells/well) and LLC cells (7.03 × 10^4^ cells/well) were plated in 96-well plates (Cat. No. 3595; Corning, Corning, NY, USA) and incubated for 22 h. The cells were then washed once with PBS+/+ and incubated at 37°C for 80 min with 50 µL of an infectious suspension containing rSFV particles at MOI = 15 (defined as 15 infectous units in BHK-21 cells) diluted in PBS+/+. The infectious suspension was then replaced with normal BHK-21 or LLC growth medium. Cells incubated in PBS+/+ without viral particles were used as a negative control. Cell morphology was observed 47 h after infection using bright-field microscopy, and 1 μCi of [methyl-^3^H]-thymidine (^3^H-TdR; Cat. No. MT6032; Hartmann Analytic GmbH, Braunschweig, Germany) per 1 mL of cell growth medium was then added. After a further 24 h of incubation, the cells were subjected to two freeze/thaw cycles and harvested using a Tomtec-96 cell harvester (Tomtec, Hamden, CT, USA) onto fiberglass filters (Cat. No. 1450-421; PerkinElmer Inc., Waltham, MA, USA). Cell growth was determined by measuring the incorporation of ^3^H-TdR into proliferating cells as counts per minute (cpm) using a microplate scintillation counter (MicroBeta Trilux 1450, PerkinElmer Inc.).

### Production of SFV Vector-Encoded Cytokines

To produce rSFV-encoded TNF-α and IFN-γ, BHK-21 cells were cultivated in 75 cm^2^ cell culture flasks (Cat. No. CLS430641; Sigma-Aldrich) until 80% confluency, washed once with PBS+/+ and then incubated at 37°C for 80 min with 5 mL of a suspension containing 1.92 × 10^8^ IFU of SFV-Tnfa or 1.21 × 10^8^ IFU of SFV-Ifng in PBS+/+. The virus-containing suspensions were then replaced with BHK-21 cultivation medium containing 1% FBS. After 36 h, the cell growth medium was harvested and centrifuged at 300 *g* for 5 min to remove the cells before the supernatants were transferred to a new tube, which was then centrifuged at 11,000 *g* for 8 min. The remaining supernatants were stored at −80°C and used as a source of vector-derived (vd) TNF-α or vdIFN-γ. The concentrations within the supernatants of the cytokines vdTNF-α or vdIFN-γ were 332.28 and 795.92 ng/mL, respectively. The levels of cytokines within the supernatants were quantified using Luminex bead-based assay with Bio-Plex Mouse Cytokine Group I IFN-γ or TNF-α sets (Cat. No. 171G5017M and 171G5023M; Bio-Rad, Hercules, CA, USA) according to the manufacturer’s instructions.

### Secretion of SFV Vector-Encoded Cytokines from Cancer Cells

Baby hamster kidney and LLC cells were plated in 24-well plates (Cat. No. 142475; Thermo Fisher Scientific) and cultivated until the next day when the cells had reached 100 and 50% confluency, respectively. The cells were washed once with PBS+/+, and 600 µL of infectious suspension containing either SFV-Tnfa or SFV-Ifng particles at a MOI = 1, MOI = 10 or MOI = 40 (calculated according to the titration in BHK-21 cells) in PBS+/+ was added per well. After cells were incubated with the infectious suspension for 80 min at 37°C, the infectious suspension was discarded, and 500 µL of fresh cell growth medium was added per well. The cells were cultured for 24 h before the growth medium was collected and centrifuged for 6 min at 300 *g*. The supernatant was then transferred to a new tube, which was centrifuged at 11,000 *g* for 10 min. The supernatants were stored at −80°C, and the levels of secreted vdTNF-α and vdIFN-γ were determined using Luminex bead-based assay with Bio-Plex Mouse Cytokine Group I IFN-γ and TNF-α sets (Cat. No. 171G5017M and 171G5023M; Bio-Rad) according to manufacturer’s recommended protocols.

### Induction of Cell Death in L929 Fibroblasts and LLC Cancer Cells by TNF-α and IFN-γ

L929 fibroblasts or LLC cells were plated in 24-well plates (Cat. No. 142475; Thermo Fisher Scientific) and cultivated in RPMI 1640 containing 10% FBS (Sigma-Aldrich) for approximately 24 h until the cells reached 50–90% confluency. To verify the functional activity of vdTNF-α, it was added to L929 cells in parallel with recombinant (r)TNF-α (Cat. No. 315-01A; PeproTech, Rocky Hill, NJ, USA) to reach a final concentration of 6.6, 20, or 60 ng/mL, and the cells were then cultivated for 24 h. L929 cells that were treated with 1 µM staurosporine (Cat. No. S4400; Sigma-Aldrich) for 24 h were used as the positive control for apoptosis induction (65), and untreated cells were used as the negative control. LLC cells were treated with either 50 ng/mL rTNF-α or 100 ng/mL rIFN-γ for 48 h to test cytotoxicity of cytokines to LLC cancer cells. LLC cells were also treated with 50 ng/mL rTNF-α in combination with 100 ng/mL rIFN-γ for 24 h or 48 h. LLC cells were pretreated with 100 ng/mL rIFN-γ for 24 h followed by 24 h treatment with 50 ng/mL rTNF-α. Untreated cells were used as the negative control, whereas L929 cells that were treated with 50 ng/mL of rTNF-α were used as the positive control for apoptosis induction. After treatments L929 or LLC cells were detached using 0.25% trypsin-EDTA and centrifuged before they were labeled with annexin V-FITC and PI using an Apoptosis Detection Kit (Cat. No. 556547; BD Biosciences) according to the manufacturer’s protocol. Briefly, the cell pellet was resuspended in 100 µL 1 x annexin V binding buffer and stained using 1 µg/mL annexin V-FITC and 5 µg/mL PI by incubating the cells at RT for 15–20 min. Then, 400 µL of additional annexin V binding buffer was added. The cell suspension was immediately analyzed using flow cytometry in a BD LSRFortessa instrument (BD Biosciences). The data were analyzed using FlowJo V10 software.

### Verification of the Functional Activity of vdIFN-γ Using a Cancer Cell Growth Inhibition Assay

Murine BMDMs (4 × 10^6^ cells/dish) were seeded in 90-mm nontreated cell culture dishes (Cat. No. 734-2359; VWR) and cultivated in complete BMDM growth medium (RPMI-1640 with 10% FBS (Biochrom) and 10% L929-CM) for 2–3 days. The BMDMs were detached by incubating the cells in cold PBS−/− at +4°C for 15–20 min and then harvested *via* centrifugation for 6 min at 300 *g*. To block cell proliferation, the BMDMs were incubated with 10 µg/mL mitomycin C (Cat. No. M4287; Sigma-Aldrich) at 37°C for 2 h. The cells were then washed twice with PBS−/− and plated in triplicate at one of three different densities (i.e., 6 × 10^4^, 3 × 10^4^, and 3 × 10^3^ cells/well) in 96-well plates in 200 µL of macrophage growth medium. The cells were then cultivated for 24 h. To stimulate the macrophages, we treated them with 100 ng/mL of the toll-like receptor (TLR) 2/1 heterodimer agonist Pam3CSK4 (referred to as Pam3; Cat. No. tlrl-pms; InvivoGen, San Diego, CA, USA), 100 ng/mL of recombinant (r)IFN-γ (Cat. No. 315-05; PeproTech) or 100 ng/mL of vdIFN-γ for 24 h. Untreated BMDMs served as the negative control, and BMDMs that were treated with 100 ng/mL of Pam3 in combination with 100 ng/mL of rIFN-γ were used as the positive control. Some BMDMs were treated for 24 h at 37°C with 100 ng/mL of Pam3 in combination with fourfold dilutions of rIFN-γ at concentrations ranging from 0.0015 to 100 ng/mL. Alternatively, other BMDMs were treated with 100 ng/mL of Pam3 in combination with 16-fold dilutions of vdIFN-γ at concentrations ranging from 0.0015 to 100 ng/mL. After 24 h of treatment, 100 µL of the cell culturing medium was replaced with 100 µL of a LLC cell suspension containing 3 × 10^4^ cells/mL to form co-cultures of macrophages and cancer target cells. The resulting macrophage:cancer cell ratios were 20:1, 10:1, and 1:1. BMDMs that were incubated alone served as the negative control for macrophage growth, and LLC cells that were incubated alone served as the positive control for normal LLC cell growth. After 20 h, ^3^H-TdR was added to all the wells at a concentration of 1 μCi per 1 mL of growth medium, and the cultures were incubated for an additional 24 h. Next, the plates were submitted to two freeze/thaw cycles and then harvested using a Tomtec-96 cell harvester onto fiberglass filters. Cell growth was analyzed by measuring the incorporation of ^3^H-TdR into proliferating cells as cpm using a microplate scintillation counter (MicroBeta Trilux 1450).

### Determination of Nitric Oxide (NO) Production by Activated Macrophages

Nitrite (${\text{NO}}_{2^{-}}$) levels were measured using the Griess test in cultivation medium harvested from BMDMs at 24 h post-treatment. Cultivation medium (100 µL) was obtained from the wells with the highest densities of BMDMs and transferred to new 96-well round-bottom plates (Cat. No. 3799; Sigma-Aldrich). These were centrifuged for 6 min at 400 *g*. Then, 50 µL of the supernatant was pipetted into new 96-well flat-bottom plates (Cat. No. 3595; Corning), and an equal volume of a solution containing 1% sulfanilamide (Cat. No. S9251; Sigma-Aldrich) and 5% phosphoric acid (Cat. No. 345245; Sigma-Aldrich) in H_2_O was added. The plates were incubated in the dark for 10 min at RT to allow the sulfanilic acid to convert to diazonium salt. After the incubation period, 50 µL of 0.1% *N*-(1-naphthyl)ethylenediamine (NED; Cat. No. N9125; Sigma-Aldrich) in H_2_O was added to the samples, standards, and blank to induce the immediate formation of azo dye. Solutions of sodium nitrite (NaNO_2_) in twofold dilutions at concentrations ranging from 3.13 to 100 µM were used as the standard and set up in triplicate. Water was used as the blank. Absorbance was measured at 540 nm using a microplate spectrophotometer (Epoch; BioTek Instruments, Winooski, VT, USA).

## Results

### Construction of SFV-Based Vectors and Production of Recombinant Viral Particles

**Figure 1:**
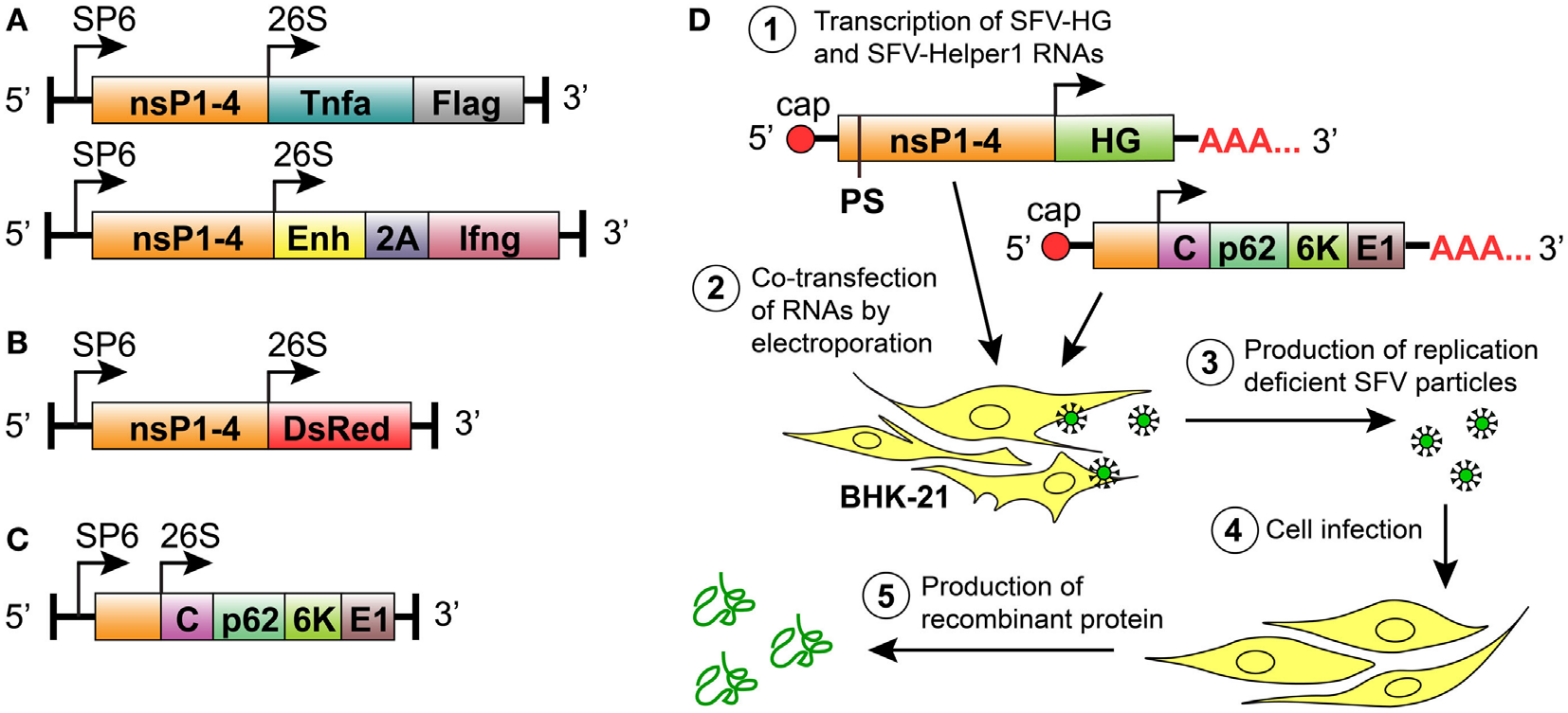
Semliki Forest virus (SFV) vectors used in this study for replication-deficient expression system. All vectors comprised the prokaryotic SP6 RNA polymerase promoter for *in vitro* transcription of the recombinant SFV replicons. **(A)** Replication-deficient vectors encoding SFV non-structural protein genes (nsP1–4) and the heterologous genes (HGs) *Tnfa* or *Ifng* downstream of the SFV 26S subgenomic promoter. The mouse TNF-α gene (*Tnfa*) was followed by a FLAG-tag-encoding sequence. The mouse IFN-γ gene (*Ifng*) was inserted in frame with the minimal capsid translation enhancer sequence (*Enh*) using the 2A auto-protease sequence (*2A*) from foot and mouth disease virus as a linker. **(B)** Replication-deficient reporter construct consisting of SFV nsP1–4 and the fluorescent protein-encoding gene *Ds-Red* downstream of an SFV 26S subgenomic promoter. **(C)** The SFV helper vector SFV-Helper1, which encodes the full length of the structural protein ORF (i.e., C, capsid protein; p62, 6K and E1, and envelope proteins) downstream of an SFV 26S subgenomic promoter. **(D)** Production of recombinant particles and proteins by the replication-deficient SFV vector system: (1) *in vitro* transcription of RNA encoding the HG and the nsP1–4, and the transcription of helper RNA carrying SFV structural genes. The packaging signal (PS) for the selective encapsidation of the RNA encoding nsP1-4 and the HG is present in the nsP2 region of SFV-HG RNA. Both RNAs are capped at the 5-prime end and polyadenylated at the 3-prime end; (2) BHK-21 cells are transfected with SFV-HG and SFV-Helper RNAs by electroporation; (3) BHK-21 cells produce replication-deficient SFV particles carrying HGs; (4) target cells are infected with replication-deficient SFV particles; and (5) the heterologous protein is produced at high levels without production of new viral particles.

We constructed two new replication-deficient SFV-based vectors that encoded either mTNF-α or mIFN-γ (Figure 1A). To express mTNF-α in the SFV vector, a sequence containing the *Tnfa* gene followed by a Flag-tag-encoding sequence at the 3-prime end was subcloned into the pSFV1-NruI vector (Figure 1A). The pSFV-Ifng vector was made by inserting the mIFN-γ gene (*Ifng*) into the pSFV1/Enh vector downstream and in-frame with the SFV capsid translation enhancer sequence (*Enh*) that was fused to the 2A auto-protease sequence of foot and mouth disease virus (FMDV) (Figure 1A). The 2A-encoding sequence was included to induce the cleavage of the vdIFN-γ protein from the 34 amino acid-long peptide that was translated from the *Enh* sequence. It has been shown that by incorporating an enhancer element within SFV vector, the expression of the heterologous protein can be increased by up to 10-fold due to enhanced translation (7, 66). In contrast to the SFV-Ifng construct, we did not incorporate the enhancer element into the SFV-Tnfa vector aiming to retain low levels of translated TNF-α if necessary since lower levels of TNF-α might be important for normalization of tumor vasculature (46). The previously constructed vector pSFV-DsRed, which encode a fluorescent protein (Figure 1B), was used as a control to detect cell infection. All vectors comprised the prokaryotic SP6 RNA polymerase promoter for *in vitro* transcription of recombinant SFV replicons, as well as the vectors encoded SFV replicase complex, which consists of non-structural proteins (nsP) 1–4 (Figures 1A–C). The SFV structural proteins C, p62, 6 K, and E1, which are necessary for the formation of infectious virus particles, were encoded by the SFV-Helper1 vector (Figures 1C,D). The heterologous genes (HGs) and the genes encoding the structural SFV proteins were inserted downstream of the SFV 26S promoter (Figures 1A–C). In this study, replication-deficient rSFV particles were produced by BHK-21 cells that were co-transfected with RNA transcribed from the SFV-Helper1 vector and RNA transcribed from a vector encoding a HG (*Tnfa, Ifng*, or *DsRed*) (Figure 1D). The rSFV viral particles were quantified in BHK-21 cells and revealed titers ranging from 10^7^ to 10^8^ IFU/mL. More specifically, the mean viral titers were 1.15 × 10^8^ IFU/mL of SFV-Tnfa, 7.23 × 10^7^ IFU/mL of SFV-Ifng, and 1.46 × 10^8^ IFU/mL of SFV-DsRed. The SE of the mean viral titers between decuplicates did not exceed 10%.

### Mouse and Human Lung Carcinoma Cells Are Susceptible to rSFV Infection, While Macrophages Are Resistant

**Figure 2:**
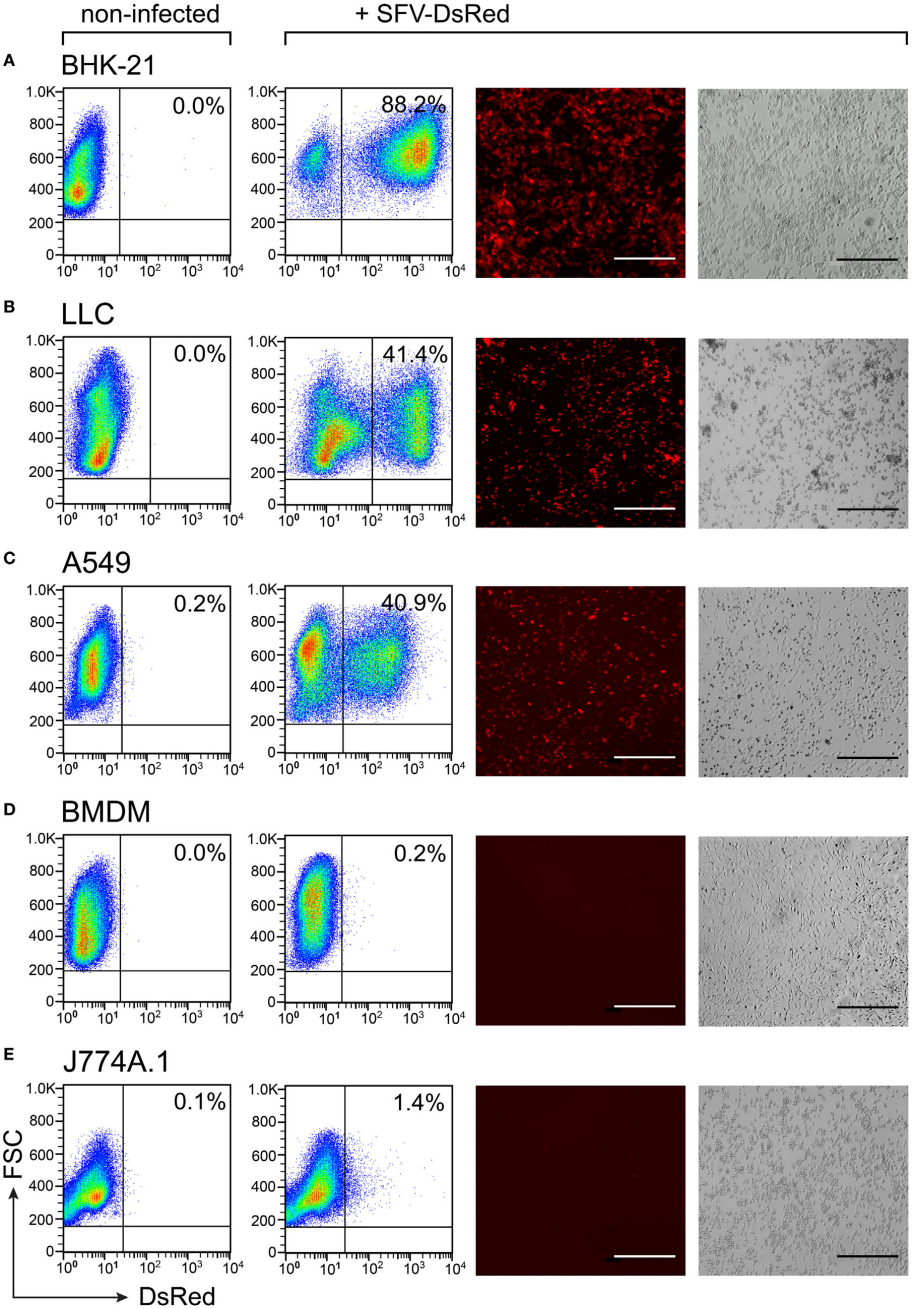
Susceptibility to Semliki Forest virus (SFV) infection varies between cell types. Cells were plated and infected with SFV-DsRed particles at MOI = 15 [determined using baby hamster kidney (BHK-21) cells] the next day. DsRed expression was evaluated at 24 h post-infection using flow cytometry and fluorescence microscopy. Non-infected cells were used as a negative control. From left to right: flow cytometry data for DsRed expression in non-infected and infected samples with the numbers representing the percentage of DsRed-positive cells as the mean of duplicates; and fluorescence microscopy images are shown with the corresponding bright-field microscopy images (scale bars, 50 µm). **(A)** The hamster fibroblast cell line BHK-21 was used as a positive control; **(B)** mouse lung carcinoma cell line Lewis lung carcinoma (LLC), **(C)** human lung carcinoma cell line A549, **(D)** mouse bone marrow-derived macrophages (BMDMs), and **(E)** mouse macrophage cell line J774A.1. The experiment was repeated three or more times with the SE not exceeding 10% between independent experiments. The average of duplicates with SEM <10% from one representative experiment is shown.

We wanted to use rSFV to deliver cytokine genes into cancer cells for subsequent production of the tumor-suppressive factors. Cell susceptibility to viral infection is a prerequisite for successful gene delivery into cells using a viral vector. We investigated the ability of rSFV particles to infect both cancer cells and macrophages using the reporter vector SFV-DsRed. We infected cells with SFV-DsRed at a concentration 15 virus particles per cell (i.e., MOI = 15). We observed the expression of the DsRed fluorescent protein using fluorescence microscopy and quantified the DsRed-expressing cells using flow cytometry at 24-h post-infection (Figures 2A–E). In all the tested cell types, non-infected cells were used as the negative control for DsRed expression. The BHK-21 cell line was used as a positive control due to being highly susceptible to SFV infection, as demonstrated by flow cytometry analysis that showed that 88.2% of the BKH-21 cells were infected under the experimental conditions used in this study (Figure 2A). We verified that both the murine (LLC) and the human (A549) lung carcinoma cell lines were infected by the rSFV particles because 41% of both cell types were DsRed-positive (Figures 2B,C). However, we also found that neither the mouse BMDMs nor the murine macrophage cell line J774A.1 was susceptible to SFV because less than 1.5% of the cells were DsRed-positive (Figures 2D,E). Macrophages remained resistant to SFV infection also when we increased the concentration of SFV particles during infection (data not shown).

**Figure 3:**
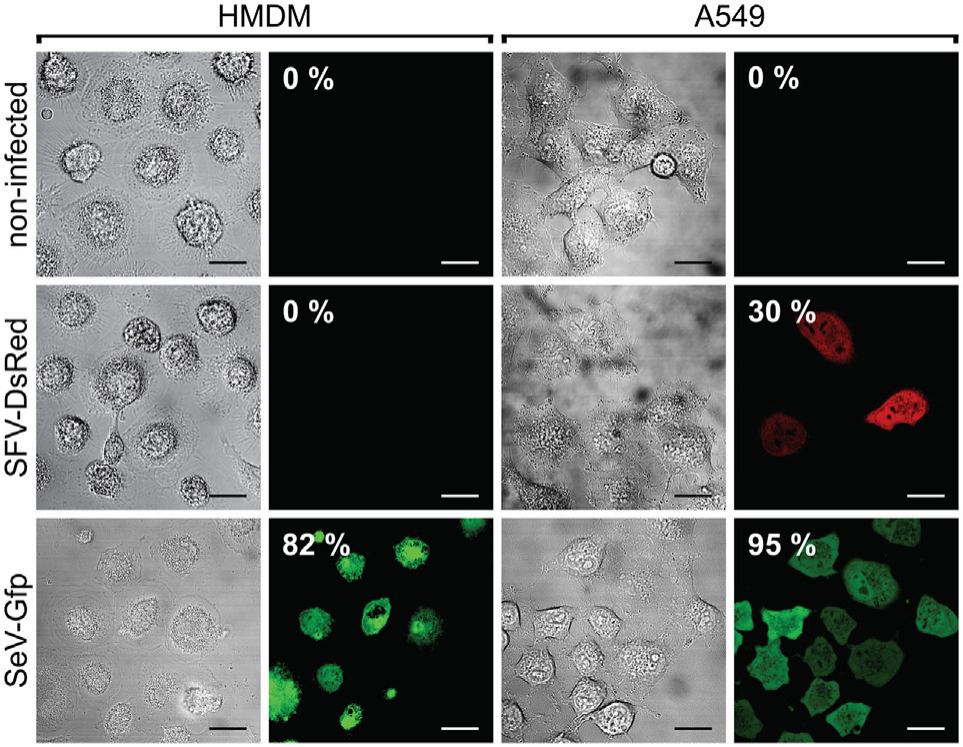
Human macrophages are resistant to Semliki Forest virus (SFV) infection. Human monocyte-derived macrophages (HMDMs), and the human lung carcinoma cell line A549 were infected with either SFV-DsRed or SeV-Gfp virus particles. The resulting cell monolayers were analyzed using phase-contrast microscopy, and the expression of DsRed and GFP was evaluated using fluorescence microscopy 24 h post-infection (scale bars, 20 µm). The numbers in the images indicate the percentages of infected cells. Non-infected cells were used as negative controls. HMDMs were not susceptible to infection with SFV-DsRed, whereas A549 cells were infected with SFV, as expected (shown in the second row). Sendai virus (SeV)-Gfp virus particles were used as a positive control because they are capable of infecting both HMDMs and A549 cells, as shown in the third row. Total HMDM resistance to SFV infection was confirmed in three independent experiments using HMDMs from different donors. Data from one representative experiment are shown where the percentages represent average fluorescent protein-expressing cell population with SEM <5%.

To confirm these findings in human macrophages, we differentiated macrophages from blood-derived monocytes and infected the cells with SFV-DsRed virus particles at a MOI = 15 (calculated according to the titer that was determined in BHK-21 cells). The SeV is known to infect human macrophages (67), and we therefore included a SeV that encoded GFP (SeV-Gfp) at a MOI = 15 as a positive control. In parallel, we infected the human lung carcinoma cell line A549 with both types of recombinant viruses at a MOI = 15, and used the infected A549 cells as a positive control for SFV infection. To calculate the percentages of infected cells, we counted the total number of cells as well as the number of fluorescent protein-expressing cells within the same viewfields (multiple viewfields per sample) using phase-contrast and fluorescence microscopy at 24 h post-infection. In these experiments, 30% of the positive control sample (the human cancer cell line A549) was infected with SFV-DsRed, whereas the HMDMs remained uninfected (Figure 3, middle panel). Contrary to our findings for SFV, SeV-Gfp infected both HMDMs and A549 very efficiently, with 82 and 95% of the HMDMs and A549 cells, respectively, being positive for GFP (Figure 3, lower panel). Taken together, these data show that lung carcinoma cells are efficiently infected by rSFV, whereas primary mouse and human macrophages are resistant to SFV infection, suggesting that rSFV may be used to deliver genes for cancer therapy without killing tumor-associated macrophages.

### Macrophages Remain Viable after Challenge with SFV Particles

We wanted to test macrophage viability after challenge with rSFV particles. Both J774A.1 macrophages and LLC cancer cells were subjected to rSFV-DsRed particles at MOI = 10 during 80-min infection protocol. Cell infection rate was determined 48 h post-infection by quantifying DsRed-expressing cells using flow cytometry. Cells were also stained with annexin V-FITC and DAPI followed by flow cytometry analysis to determine cell viability.

**Figure 4:**
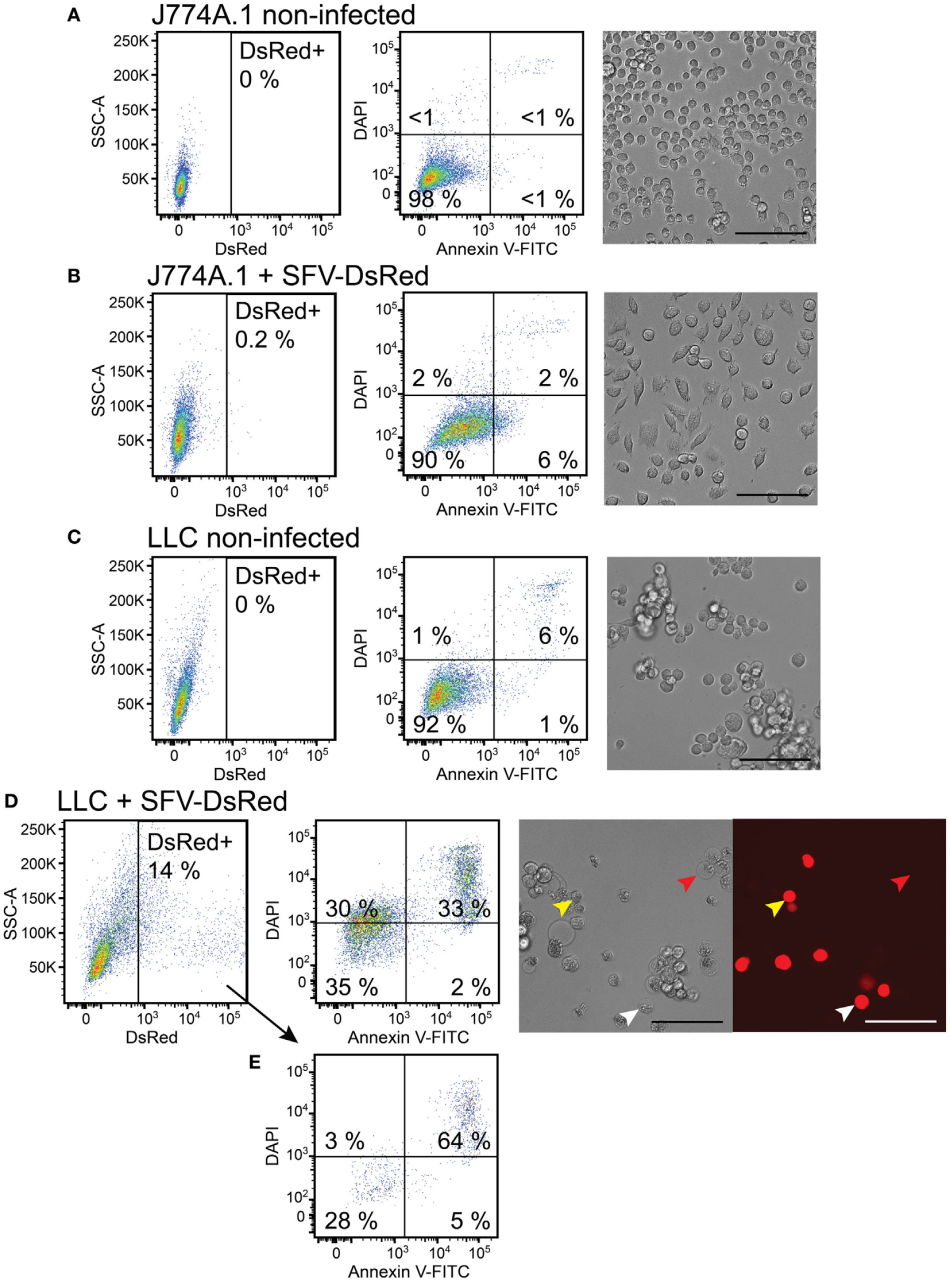
Macrophages remain viable, whereas cancer cells undergo cell death after challenge with Semliki Forest virus (SFV). J774A.1 mouse macrophages and Lewis lung carcinoma (LLC) carcinoma cells were infected with SFV-DsRed particles at MOI = 10 [determined using baby hamster kidney (BHK-21) cells]. DsRed expression (first column, *x*-axis) was evaluated at 48 h post-infection using flow cytometry and fluorescence microscopy (scale bar 100 µM). Cell morphology was observed using bright-field microscopy (scale bar 100 µM) and cell death was quantified 48 h post-infection using flow cytometry analysis after cell staining with annexin V-FITC (second column, *x*-axis) and DAPI (second column, *y*-axis). Annexin V-positive/DAPI-negative cells were regarded as early apoptotic, whereas annexin V-positive/PI-positive cells were regarded as late apoptotic and necrotic. **(A)** Non-infected J774A.1 macrophages were used as a negative control. **(B)** J774A.1 macrophages were resistant to SFV infection, stayed viable but changed their morphology 48 h after challenge with SFV. **(C)** Non-infected LLC cells were used as a negative control. **(D)** LLC cells were susceptible to SFV infection and underwent cell death. DsRed expression was observed using microscopy in both apoptotic (white arrows) and viable (yellow arrows) cells, whereas some apoptotic cells lacked DsRed expression (red arrows). **(E)** The infected DsRed-positive cells were both viable and undergoing cell death. The average of duplicates with SEM <10% from one experiment is shown.

As expected, we observed that 98% of non-infected macrophages stayed viable after 48 h as determined using flow cytometry and retained round morphology, which is characteristic to J774A.1 cells in normal culture conditions (Figure 4A). Also 48 h after challenge with rSFV-DsRed particles 90% of macrophages remained viable, whereas 6% of macrophages were single-positive for Annexin V (Figure 4B). Interestingly, light microscopy revealed that macrophages changed morphology 48 h after challenge with rSFV by gaining spindle-like or polygonal shape (Figure 4B). LLC cells were used in parallel as a positive control for cell infection. As expected, the non-infected LLC cells showed 92% viability and were characterized by mixed population of adherent and floating cells with round or spindle-like morphology (Figure 4C). However, the infected LLC cells underwent cell death 48 h after infection with rSFV, and only 35% of LLC cells were viable (Figure 4D). We observed that 33% of rSFV-challenged LLC cells were double-positive for Annexin V-FITC and DAPI suggesting apoptotic/necrotic cell death. A LLC population of 30% was single-positive for DAPI 48 h after infection suggesting affected cell membrane permeability (Figure 4D). Light microscopy revealed that LLC cells possess apoptotic morphology characterized by round swollen cells with translucent cytoplasm after infection with rSFV (Figure 4D). We observed microscopically that both DsRed-positive (infected) and DsRed-negative (non-infected) cells undergo apoptosis after challenge with rSFV particles (Figure 4D, red and white arrows), whereas part of infected DsRed-expressing cells remain viable 48 h post-infection (Figure 4D, yellow arrows). Flow cytometry analysis revealed that 69% of all DsRed-expressing cells undergo cell death characterized mostly by being double-positive for Annexin V-FITC and DAPI (Figure 4E). Our data show that macrophages are not only resistant to SFV infection but also remain viable after challenge with rSFV particles.

### SFV Infection Inhibits Growth of Murine Lung Carcinoma Cells

**Figure 5:**
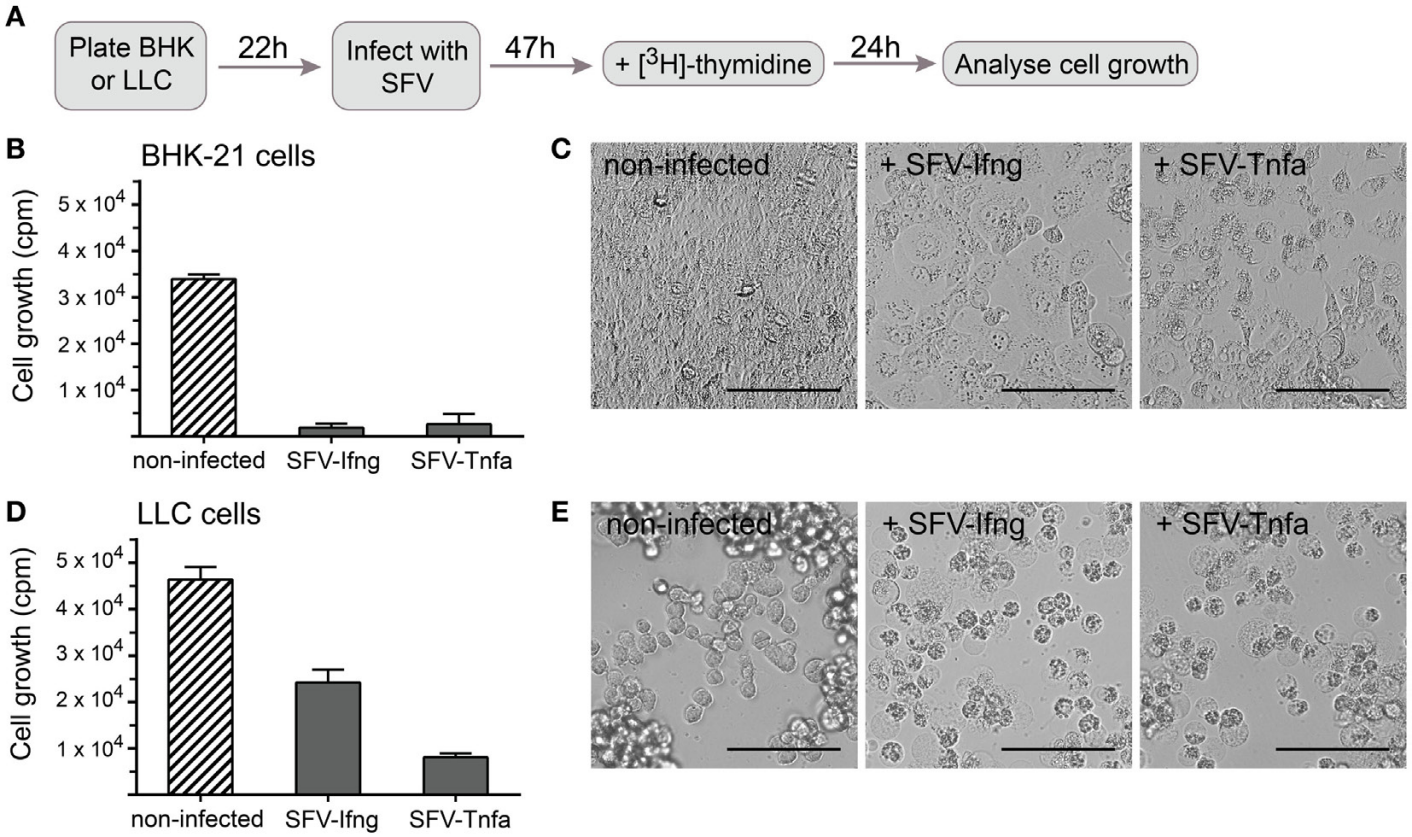
Cancer cell growth is inhibited by infection with Semliki Forest virus (SFV) particles. **(A)** Schematic overview of the experiment. Baby hamster kidney (BHK-21) or Lewis lung carcinoma (LLC) cells were plated and incubated for 22 h before they were infected with SFV-Ifng or SFV-Tnfa particles at MOI = 15. The cells were incubated for 47 h after infection, and [^3^H]-thymidine was then added to detect proliferating cells. After 24 h, the cells were harvested, and cell growth was determined by measuring the incorporated [^3^H]-thymidine as counts per minute (cpm, depicted on the *y*-axis). **(B)** As expected, infecting BHK-21 cells with SFV resulted in substantial growth inhibition. **(C)** BHK-21 cells underwent cell death 47 h post-infection as observed using bright-field microscopy (scale bars, 50 µm). **(D)** LLC cell growth was inhibited by infection with different SFV particles. **(E)** LLC cells underwent cell death 47 h post-infection as observed using bright-field microscopy (scale bars, 50 µm). Two experiments were performed in triplicates with SE not exceeding 10% between independent experiments. The bars represent the mean values of triplicates ± SEM from one representative experiment.

It has been shown that SFV infection induces cellular death *via* p53-independent apoptosis (21). To determine whether rSFV has a direct effect on cancer cell growth, we used *in vitro* assay to evaluate proliferation of infected LLC cells. The cells were cultivated until approximately 70–80% confluency, and they were then infected with SFV-Ifng or SFV-Tnfa viral particles at MOI = 15. To determine cell growth, a radioactive thymidine, which incorporates into new DNA strands during cell division, was added to the cell cultures at 47 h post-infection. The experimental setup is illustrated in Figure 5A.

BHK-21 cells were used in parallel as a positive control for efficient cell infection by SFV and showed nearly complete growth inhibition (Figure 5B). Growth inhibition was similar independent on whether SFV carried a gene encoding TNF-α or IFN-γ (Figure 5B). It was observed using light microscopy that BHK-21 cells lost confluency and gained apoptotic cell morphology characterized by translucent cytoplasm, granularity, or round shape 47 h post-infection (Figure 5C). LLC cell growth was inhibited by half 47 h after infection with SFV-Ifng and by 80% after infection with SFV-Tnfa (Figure 5D). Light microscopy revealed that LLC cells acquired apoptotic cell morphology characterized by round shape, granularity, and translucent cytoplasm 47 h after infection with either SFV-Ifng or SFV-Tnfa viral particles (Figure 5E). The data show that rSFV particles encoding murine IFN-γ or TNF-α efficiently inhibited LLC cell growth at 47 h post-infection.

### Mouse Lung Carcinoma Cells Produce and Secrete SFV-Encoded TNF-α and IFN-γ

**Figure 6:**
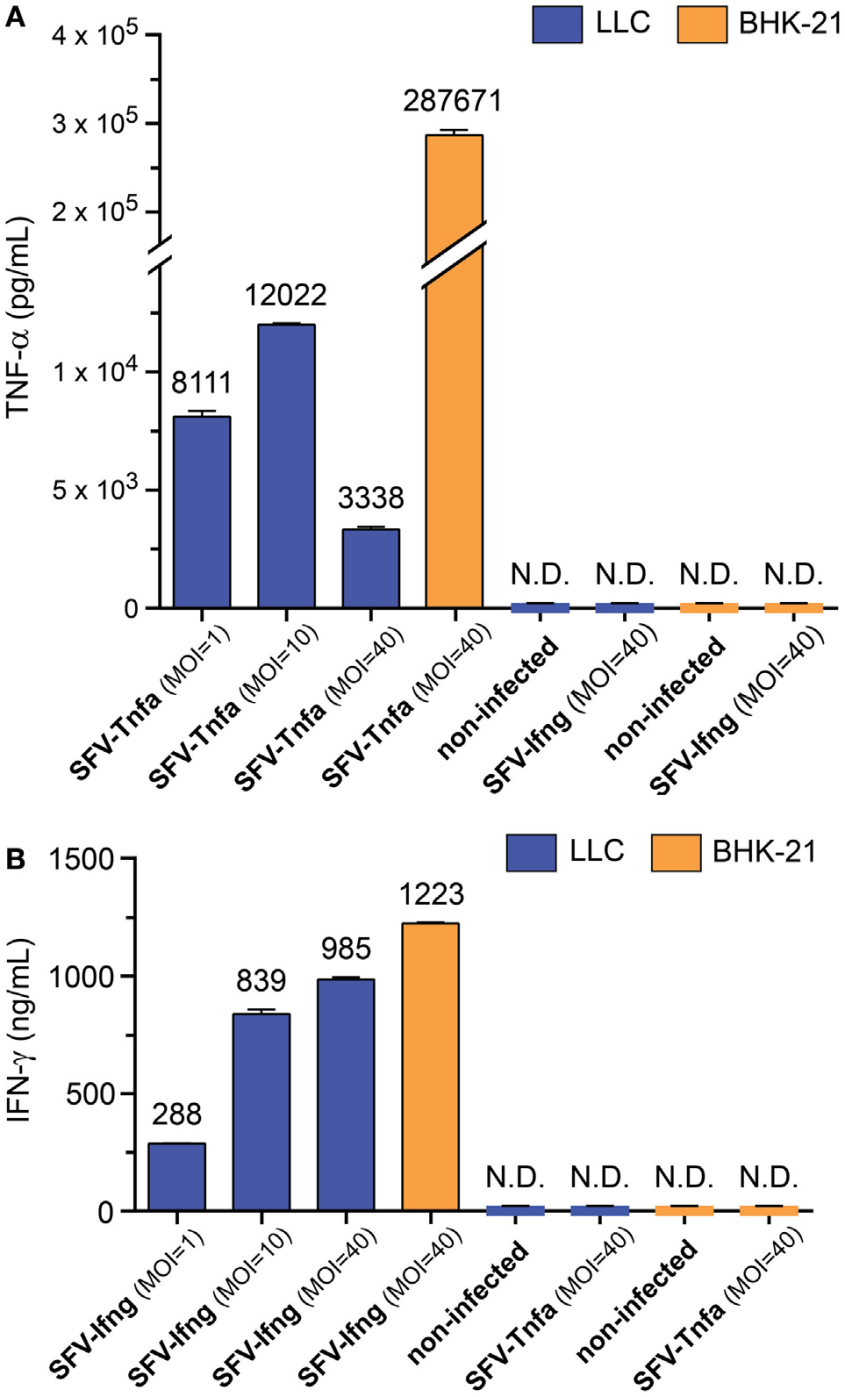
Semliki Forest virus (SFV)-encoded cytokines are produced and secreted by cancer cells. Baby hamster kidney (BHK-21) and Lewis lung carcinoma (LLC) cells were infected with either SFV-Ifng or SFV-Tnfa at MOI = 1, MOI = 10, or MOI = 40 (determined using BHK-21 cells). The level of secreted **(A)** TNF-α or **(B)** interferon (IFN)-γ in the cell culture medium was determined using Luminex bead-based assay at 24 h after infection and is shown on the *y*-axis. The cytokines were undetectable in both the negative controls of non-infected cells and the cultures that were infected with a mismatched SFV that did not encode the cytokine of interest. N.D.—not detectable. Two independent experiments were performed in duplicates. Data from one representative experiment is shown. The bars represent the mean values of duplicates ± SEM.

In the next set of experiments, we sought to determine whether LLC cells secrete cytokines after infection with SFV-Tnfa and SFV-Ifng virus particles. In this experiment, BHK-21 cells were used as a positive control. The LLC and BHK-21 cells were cultivated until 50 and 100% confluency, respectively, before they were infected with SFV-Tnfa or SFV-Ifng at a MOI = 1, MOI = 10, or MOI = 40 (calculated according to the titration in BHK-21 cells). After 24 h, the levels of secreted vdIFN-γ and vdTNF-α in the cell cultivation medium were analyzed using Luminex bead-based assay. LLC cells infected with SFV-Tnfa secreted vdTNF-α (range, 10^3^–10^4^ pg/mL for all tested MOIs) (Figure 6A). There was no clear correlation between the MOI of the virus particles and the level of vdTNF-α (Figure 6A). The vdTNF-α was not detected in the medium of non-infected cells or in the medium of cells infected with SFV-Ifng, confirming that the secreted vdTNF-α was derived from the *Tnfa* gene that was delivered using SFV (Figure 6A).

In LLC cells, infection with SFV-Ifng resulted in the secretion of vdIFN-γ into the growth medium at a concentration ranging from 2.9 to 9.9 × 10^2^ ng/mL at 24 h after infection (Figure 6B). The concentration of vdIFN-γ appeared to correlate with the concentration of viral particles, and the highest tested MOI resulted in the highest level of vdIFN-γ (Figure 6B). The vdIFN-γ was not detected in media obtained from non-infected cells or from cells infected with SFV-Tnfa, indicating that the vdIFN-γ originated from the SFV-delivered cytokine gene (Figure 6B). In conclusion, these results show that both BHK-21 and LLC cells produce and secrete vdTNF-α and vdIFN-γ when infected with the rSFV vectors described in this study.

### Vector-Derived TNF-α Is Functional in Inducing Cell Death in L929 Murine Fibrosarcoma Cells

**Figure 7:**
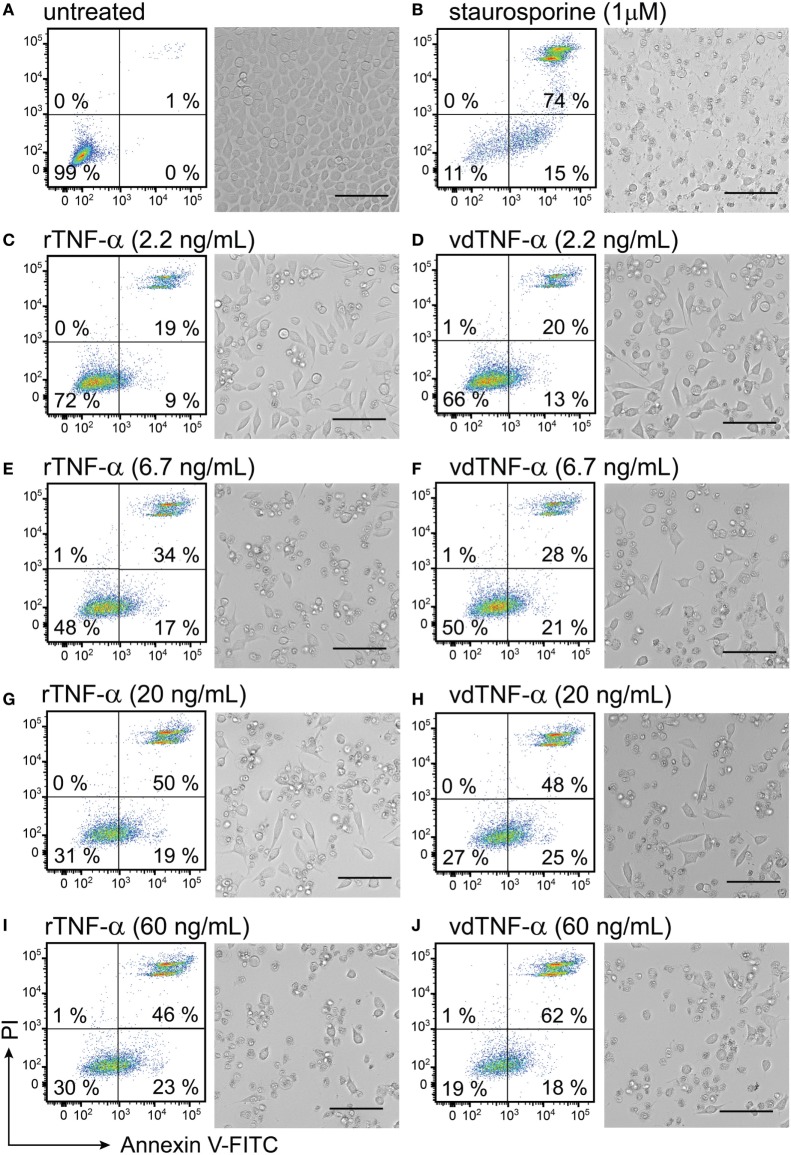
Vector-derived TNF-α induces cell death in L929 fibroblasts. L929 cells were cultured for 24 h until they reached 90% confluency. The cells were then **(A)** left untreated for 24 h and used as a negative control or **(B)** treated with 1 µM staurosporine for 24 h at 37°C and used as a positive control. The remaining cells were treated with rTNF-α or vdTNF-α for 24 h at the following concentrations: **(C,D)** 2.2 ng/mL, **(E,F)** 6.7 ng/mL, **(G,H)** 20 ng/mL or **(I,J)** 60 ng/mL. The resulting cell monolayers were analyzed using bright-field microscopy (scale bar, 50 µM). Cell death was determined using flow cytometry analysis after cell staining with annexin V-FITC (depicted on the *x*-axis) and propidium iodide (PI, depicted on the *y*-axis). Annexin V-positive/PI-negative cells were regarded as apoptotic, whereas annexin V-positive/PI-positive cells were regarded as necrotic. Two independent experiments were performed in duplicates with standard error not exceeding 10% between independent experiments. Data from one representative experiment are shown, where the percentages of the four distinct cell populations represent the averages of duplicates with SEM <10%.

To determine whether the vdTNF-α is biologically active, we utilized a known property of TNF-α which is to induce necrotic cell death in the murine fibrosarcoma cell line L929 by binding to TNF receptor-1 (68). In these experiments, L929 cells were cultivated until 90% confluency; we then added rTNF-α or vdTNF-α at concentrations varying from 2.2 to 60 ng/mL to the cells. The culture medium of BHK-21 cells infected with SFV-Tnfa particles was used as the source of vdTNF-α. At 24 h after treatment, the morphology of the L929 cells was evaluated using bright-field microscopy. Then cells were stained with annexin V-FITC to determine phosphatidylserine exposure, whereas staining with PI was used to determine loss of membrane integrity in order to quantify the numbers of viable (Annexin V−PI−), apoptotic (Annexin V+PI−), and necrotic (Annexin V+PI+) cells using flow cytometry (Figures 7A–J). Untreated L929 cells were used as a control, and viable cells were defined as those that were annexin V-negative/PI-negative (Figure 7A). Treatment with staurosporine was used as the control for apoptotic cell death (65), where annexin V-positive/PI-negative cells were regarded as early apoptotic, whereas annexin V-positive/PI-positive cells were regarded as late apoptotic undergoing secondary necrosis (Figure 7B).

In the absence of any treatment, the L929 cells reached 100% confluency after 24 h and had a normal fibroblast-like, slightly polygonal cell morphology (Figure 7A). The untreated L929 cells were also analyzed using flow cytometry and used to set the gates for the viable (Annexin V−PI−) cell population, which accounted for 99% of all events (Figure 7A). After cells were treated with 1 µM staurosporine, cell confluency was lost, and an apoptotic cell morphology characterized by cell shrinkage, a round shape and granularity was observed (Figure 7B). Only 11% of the staurosporine-treated cells were viable. The remaining cells were undergoing apoptosis and secondary necrosis (Figure 7B). In cells that were incubate with increasing concentrations of rTNF-α or vdTNF-α (2.2, 6.7, 20, or 60 ng/mL), we observed a concentration-dependent increase in the proportion of apoptotic (Annexin V+PI−) and necrotic (Annexin V+PI+) cells (Figures 7C–J) in a similar manner between rTNF-α and vdTNF-α. Treatment with vdTNF-α and rTNF-α at the lowest concentration (2.2 ng/mL) induced apoptosis in 9–13% and necrosis in 19–20% of the cells (Figures 7C,D), whereas the highest concentrations of rTNF-α and vdTNF-α (60 ng/mL) induced apoptosis in 18–23% and necrosis in 46–62% of the cells (Figures 7I,J). Moreover, after treatment with rTNF-α or vdTNF-α, we observed necrotic cell morphology characterized by round, swollen cells with translucent cytoplasm. These data verify the functionality of the vdTNF-α protein because it was able to induce apoptosis and necrosis in L929 cells in a concentration-dependent manner that was similar to that observed for rTNF-α.

### Combined Treatment of TNF-α and IFN-γ Induces Cell Death in Mouse Lung Carcinoma Cells

**Figure 8:**
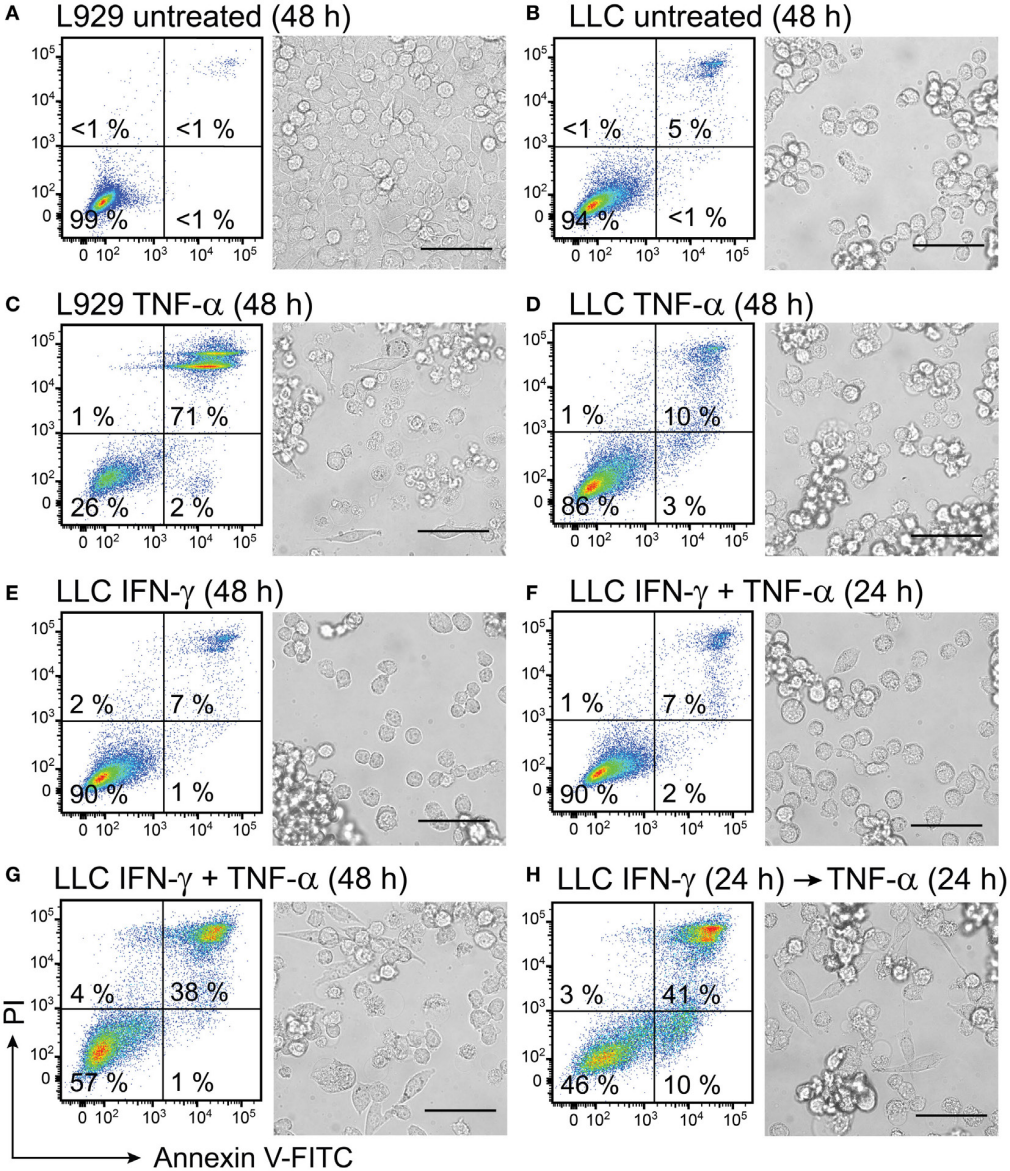
Interferon (IFN)-γ in combination with TNF-α induces cell death in mouse lung carcinoma cells. L929 fibroblasts and Lewis lung carcinoma (LLC) cancer cells were cultured for 24 h before starting treatment with recombinant cytokines. Both L929 and LLC cells were **(A,B)** left untreated for 48 h and used as negative controls or **(C)** L929 cells were treated with 50 ng/mL TNF-α for 48 h and used as a positive control. LLC cell viability was retained after treatment with **(D)** 50 ng/mL TNF-α or **(E)** 100 ng/mL IFN-γ and after **(F)** simultaneous treatment with both 50 ng/mL TNF-α and 100 ng/mL IFN-γ for 24 h. Cell death was induced in LLC cells after **(G)** simultaneous treatment with 100 ng/mL of IFN-γ in combination with 50 ng/mL of TNF-α for 48 h and after **(H)** LLC pretreatment with 100 ng/mL of IFN-γ for 24 h followed by treatment with TNF-α for 24 h. The resulting cell monolayers were analyzed using bright-field microscopy (scale bar, 50 µM). Cell death was determined using flow cytometry analysis after cell staining with annexin V-FITC (depicted on the *x*-axis) and propidium iodide (PI, depicted on the *y*-axis). Annexin V-positive/PI-negative cells were regarded as apoptotic, whereas annexin V-positive/PI-positive cells were regarded as necrotic. Experiment was performed in duplicates and repeated two times with standard error not exceeding 10% between independent experiments. One representative experiment is shown, where the percentages of the four distinct cell populations are averages of duplicates with SEM <5%.

It has been previously shown that LLC cells are resistant to cell death induced by TNF-α (69). Based on previous studies, where IFN-γ was shown to enhance TNF-α receptor expression in cancer cells (51, 52), we wanted to test whether IFN-γ may sensitize LLC cancer cells to TNF-α treatment. Therefore, we tested induction of cell death in LLC cells after combined treatment of recombinant cytokines TNF-α and IFN-γ. LLC cells were single-treated with either TNF-α or IFN-γ for 48 h. LLC cells were treated with TNF-α in combination with IFN-γ for 24 h or 48 h. LLC cells were also pretreated with IFN-γ for 24 h followed by 24 h treatment with TNF-α. L929 fibroblasts, which were treated with TNF-α, were used as a positive control. Untreated cells were used as a negative control. Cell morphology after treatments was evaluated using bright-field microscopy. Cells were stained with annexin V-FITC to determine phosphatidylserine exposure, whereas staining with PI was used to determine loss of membrane integrity in order to quantify the numbers of viable (Annexin V−PI−), apoptotic (Annexin V+PI−), and necrotic (Annexin V+PI+) cells using flow cytometry (Figures 8A–H).

In the absence of any treatment for 48 h, 99% of L929 control cells and 94% of LLC cancer cells remained viable and showed normal cell morphology (Figures 8A,B). The positive control of L929 cells, which were treated with TNF-α for 48 h, lost their confluency and showed apoptotic morphology characterized by round swollen cells with translucent cytoplasm (Figure 8C). Only 26% of the TNF-α-treated L929 cells were viable (Figure 8C). By contrast, LLC cells were resistant to treatment with TNF-α for 48 h, showed 86% viability and retained normal cell morphology (Figure 8D). Treatment with IFN-γ for 48 h was not cytotoxic to LLC cells, and 90% of the treated LLC cells remained viable (Figure 8E). Interestingly, combined treatment of LLC cells with both cytokines for 24 h also did not induce cytotoxicity in LLC cells (Figure 8F). However, a prolonged incubation of LLC cells with both cytokines for 48 h induced cytotoxicity in LLC cells resulting in 38% cells being double-positive for Annexin and PI (Figure 8G). Also pretreatment of LLC cells with IFN-γ for 24 h followed by treatment with TNF-α for 24 h was cytotoxic to LLC cells and induced apoptosis in 10% and necrosis in 41% of the cells (Figure 8H). Cell morphology of LLC cells changed after the combined treatments of LLC with both cytokines simultaneously for 48 h or with IFN-γ pretreatment followed by TNF-α (Figures 8G,H). After combined treatments with both cytokines, we observed cells with apoptotic cell morphology as well as live cells that had spread in an elongated shapes possibly due to access of free space resulting from cells dying in proximity (Figures 8G,H). All in all, combined treatment with IFN-γ and TNF-α was effective in inducing cell death in LLC cancer cells, which were resistant to single-treatments with these cytokines.

### Vector-Derived IFN-γ in Combination with a TLR2/1 Agonist Activates Macrophages toward a Cancer-Suppressive Phenotype

**Figure 9:**
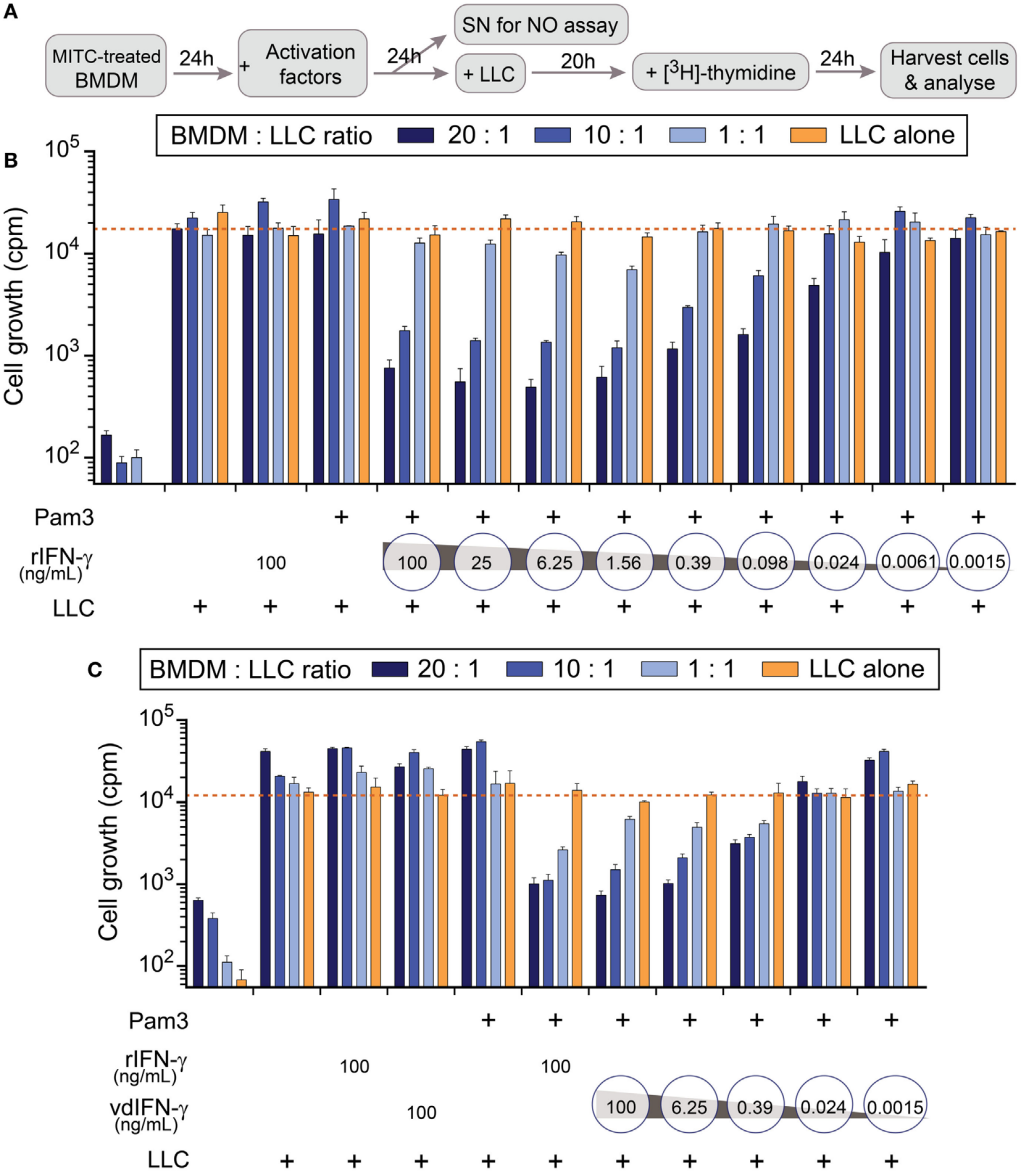
The effect of rIFN-γ or vdIFN-γ in combination with a TLR2/1 ligand on macrophage activation was detected using growth inhibition assay in Lewis lung carcinoma (LLC) cells. **(A)** Schematic timeline of the experiment. Mitomycin C-treated bone marrow-derived macrophages (BMDMs) were plated at three different densities and incubated for 24 h before macrophage-stimulating factors were added. After an additional 24 h, 100 µL of cell culture media was obtained from the wells with the highest macrophage density to measure ${\text{NO}}_{2^{-}}$ levels. LLC cells were added to the activated BMDMs, and the co-cultures were incubated for an additional 20 h. [^3^H]-thymidine was then added to the co-cultures, and after 24 h, the LLC cells were harvested to analyze cell growth by measuring the amount of incorporated [^3^H]-thymidine as counts per minute (cpm, depicted on the *y*-axis). **(B)** LLC cell growth after co-cultivation with BMDMs that were activated with Pam3 (100 ng/mL) in combination with different concentrations of rIFN-γ ranging from 100 to 0.0015 ng/mL in fourfold dilutions. Controls, from left to right: BMDMs cultivated without LLC cells, LLC cells co-cultivated with unstimulated macrophages, and LLC cells co-cultivated with macrophages that were stimulated with either rIFN-γ alone or Pam3 alone. **(C)** LLC cell growth after co-cultivation with BMDMs that were activated with Pam3 (100 ng/mL) in combination with vdIFN-γ ranging from 100 to 0.0015 ng/mL in 16-fold dilutions. Controls, from left to right: BMDMs cultivated without LLC cells, LLC cells co-cultivated with unstimulated macrophages, LLC cells co-cultivated with macrophages that were treated with either rIFN-γ, vdIFN-γ, or Pam3 at a concentration of 100 ng/mL, and LLC cells co-cultivated with BMDMs that were treated with 100 ng/mL of both rIFN-γ and Pam3. Experiments were performed in triplicates and were repeated two times with similar results. The bars represent the mean values of triplicates ± SEM from one representative experiment.

To verify that vdIFN-γ is a functional protein, we used an *in vitro* assay based on cancer cell growth inhibition mediated by activated macrophages. The macrophages were treated with mitomycin C to block their proliferation, and they were activated by applying either vdIFN-γ or rIFN-γ in combination with a second stimulus. Based on previous experiments performed in our lab, we chose to stimulate the BMDMs with IFN-γ and TLR2/1 agonist Pam3 because this combination leads to efficient inhibition of cancer cell growth (70). After the BMDMs were stimulated for 24 h, LLC cells were added, and the co-cultures were cultivated for an additional 20 h. Finally, to enable the detection of proliferating cancer cells, we added radiolabeled thymidine to the cultures at 24 h before they were harvested (Figure 9A).

In the first experiment, we treated cells with different concentrations of rIFN-γ in combination with 100 ng/mL Pam3 to identify the lowest effective dose of IFN-γ that is necessary to activate BMDMs toward a cancer-suppressive phenotype (Figure 9B). The results revealed that adding rIFN-γ within the range 100–1.56 ng/mL effectively inhibited cancer cell growth (cpm below 750 when the ratio of BMDMs:LLC cells was 20:1) in a concentration-dependent manner. Some growth inhibition was also observed at 0.098–0.39 ng/mL rIFN-γ, but minor or no effect was observed at concentrations ≤0.024 ng/mL (Figure 9B).

All controls included in this assay produced the expected results (Figures 9B,C). Treating the BMDMs with mitomycin C resulted in counts lower than 650 cpm, confirming that the treatment hindered proliferation (Figures 9B,C; first group of bars from the left). Unstimulated macrophages did not inhibit growth in LLC cells since more than 15,000 cpm were measured in the wells (Figures 9B,C; second group of bars from the left). These results were comparable to the average cpm in LLC cells that were cultivated alone (Figures 9B,C; orange line). Treating cells with either Pam3, rIFN-γ, or vdIFN-γ alone resulted in growth rates similar to those observed in LLC cells cultivated alone (Figures 9B,C).

To analyze the functionality of vdIFN-γ, we treated BMDMs with the same concentrations that were used to test rIFN-γ. The vdIFN-γ was produced by infecting BHK-21 cells with SFV-Ifng viral particles and harvesting the cell culture medium to use as a source of vdIFN-γ. The results were similar to what was observed for rIFN-γ in that incubating macrophages with vdIFN-γ in combination with Pam3 resulted in efficient cancer cell growth inhibition in a concentration-dependent manner. The lowest effective concentration of vdIFN-γ was 6.25 ng/mL, which produced less than 1,050 cpm (Figure 9C). In all the experiments, the most efficient inhibition of cancer cell growth was observed when the ratio of BMDMs to LLC cells was 20:1 or 10:1 (Figures 9B,C). Finally, the pattern of effective IFN-γ doses and the extent of growth inhibition they produced was similar between rIFN-γ and vdIFN-γ (Figures 9B,C). Based on these data, we conclude that vdIFN-γ has biological activity and can substitute for rIFN-γ.

**Figure 10:**
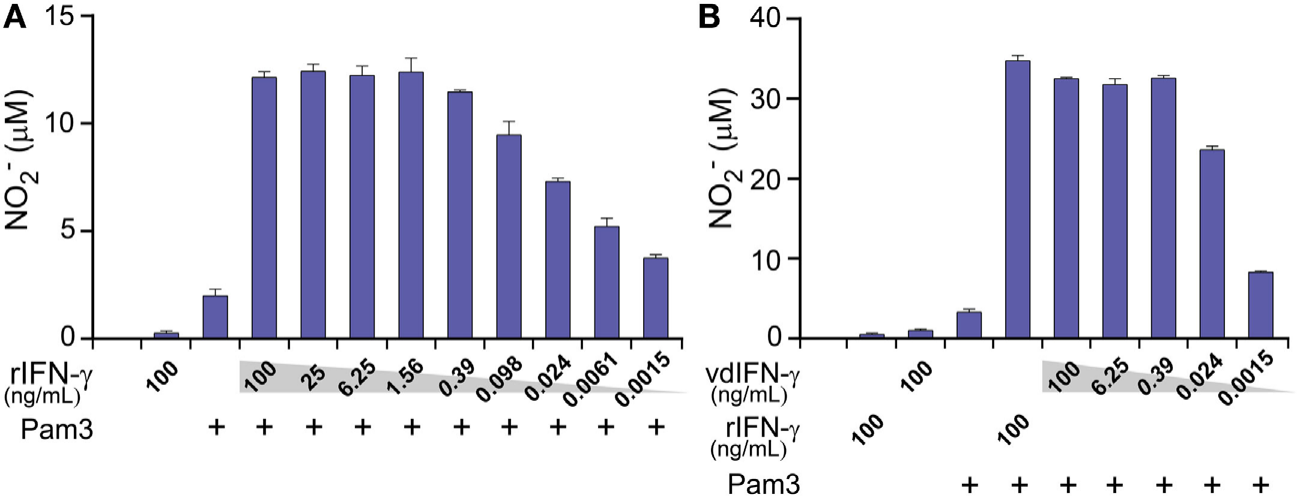
The production of nitric oxide (NO) by macrophages at 24 h after activation was determined by analyzing ${\text{NO}}_{{\text{2}}^{-}}$ levels in the cell culture medium. Mitomycin C-treated bone marrow-derived macrophages (BMDMs) were cultivated for 24 h before they were activated with the TLR2/1 agonist Pam3 (100 ng/mL) in combination with either **(A)** rIFN-γ at various concentrations ranging from 100 to 0.0015 ng/mL in 4-fold dilutions or **(B)** vdIFN-γ at concentratios ranging from 100 to 0.0015 ng/mL in 16-fold dilutions. Non-stimulated BMDMs and BMDMs that were stimulated with Pam3, rIFN-γ, or vdIFN-γ were used as the negative controls. Nitrite (${\text{NO}}_{{\text{2}}^{-}}$) levels were measured in the macrophage culture medium at 24 h post-stimulation using the Griess test. Experiments were performed in triplicates and were repeated two times with similar results. The bars represent the mean values of triplicates ± SEM from one representative experiment.

Using TLR ligands in combination with IFN-γ provides a classical set of stimuli that activates macrophages toward an M1-phenotype, which can produce NO through inducible NO synthase (70). We, therefore, determined NO production as a readout to verify that vdIFN-γ triggers macrophage activation potentially toward an M1-phenotype. We measured ${\text{NO}}_{2^{-}}$, which is the end product of the rapid oxidation of NO, in cell culture media using the Griess test. First, we verified that the BMDMs produced NO in a concentration-dependant manner at 24 h after stimulation with both rIFN-γ and Pam3 (Figure 10A). We then stimulated BMDMs with both vdIFN-γ and Pam3, and the results were similar to those observed for rIFN-γ in that the levels of ${\text{NO}}_{2^{-}}$ varied according to the concentration of vdIFN-γ (Figure 10B). The highest levels of ${\text{NO}}_{2^{-}}$ were observed when rIFN-γ and vdIFN-γ were used in a range from 0.39 to 100 ng/mL. At IFN-γ concentrations ≤0.098 ng/mL, the levels of ${\text{NO}}_{2^{-}}$ were lower (Figures 10A,B). Stimulation with rIFN-γ alone resulted in the production of only small amounts of ${\text{NO}}_{2^{-}}$ in the cell culture medium (Figure 10A). The levels of ${\text{NO}}_{2^{-}}$ were also low in cultures stimulated with vdIFN-γ alone (Figure 10B) or Pam3 alone (Figures 10A,B). Thus, IFN-γ and Pam3 had a synergistic effect on the ability of macrophages to produce NO. The higher levels of ${\text{NO}}_{2^{-}}$ that were observed following stimulation with higher doses of IFN-γ (Figures 10A,B) were well-correlated with the enhanced cancer cell growth inhibition that was observed in the growth inhibition experiments (Figures 9B,C). Taken together, data from the growth inhibition assay demonstrated that vdIFN-γ is a potent agent at very low concentrations that when combined with Pam3 induces an antitumor phenotype in macrophages that suppresses growth in LLC cells.

## Discussion

In this study, we developed two new rSFV vectors encoding either murine TNF-α or IFN-γ and showed by *in vitro* studies that the two rSFV-encoded cytokines are functional. The rSFV efficiently infected mouse and human lung carcinoma cells *in vitro*, whereas murine and human macrophages were resistant to SFV infection. The rSFV inhibited LLC cell growth, induced cancer cell death and simultaneously exploited cancer cells for production of SFV-encoded TNF-α and IFN-γ at levels that are functional *in vitro*. The functionality of SFV-encoded TNF-α was shown *via* cell death induction in L929 cells. The rSFV-encoded IFN-γ activated macrophages toward a tumoricidal phenotype *in vitro*.

Recombinant SFV has been used to express various cytokine genes in experimental tumor models resulting in therapeutic effects (2–15). For example, the same replication-deficient SFV/enh vector used in this study was previously applied for IL-12 expression in mouse breast and colon cancer models, resulting in inhibition of tumor growth upon intratumoral vector inoculation (7). Because of the antitumor potential of TNF-α (40–46) and IFN-γ (28–39) or both in combination (47–50, 53), we subcloned these cytokines in a safe replication-deficient SFV vector, which provides high and transient expression of the transgene without further virus replication (20). In order to form viral particles, we used SFV-Helper1 system (20), which encodes SFV structural proteins. We produced infectious SFV-Tnfa and SFV-Ifng recombinant virus particles in BHK-21 cells at titers 10^7^–10^8^ IFU/mL.

Various mouse and human cancer cell lines are susceptible to SFV infection although the transduction rates may differ between cell types (71–73). The hamster kidney fibroblast cell line BHK-21, which is commonly used for production of SFV particles, is efficiently infected by SFV (71). In the current study, the high susceptibility of BHK-21 cells to SFV infection was confirmed. Two cell lines of particular interest for development of lung cancer immunotherapy are mouse and human lung carcinoma cell lines, LLC and A549, respectively, which proved to be susceptible to SFV infection with a comparable transduction level of 41% 24 h post-infection for both cell lines when SFV was used at MOI = 15. This is in agreement with a previous study showing that A549 cells are susceptible to SFV infection (72). Our results encouraged us using LLC cells as target murine cancer cells further because they were successfully infected by rSFV particles and were able to express rSFV-encoded proteins.

In contrast to the tested cancer cell lines, macrophages were found to be resistant to SFV infection. Similar data were obtained with primary mouse and human macrophages, as well as with the mouse macrophage cell line J774A.1. Moreover, macrophages retained their viability after challenge with rSFV particles. The fact that macrophages are not killed by SFV but instead serve as effector cells responding to SFV-encoded factors may be an advantage for immunotherapy based on the activation of tumor-associated macrophages. Cancer cells, due to their susceptibility to SFV infection, may be exploited to produce such SFV-encoded factors. The mechanism by which macrophages remained resistant to SFV infection is unclear. The life cycle of replication-deficient rSFV particles is a several-step process comprising viral entry *via* endocytosis, a pH-dependent release of virus nucleocapsid from the endosome, capsid disassembly, viral RNA replication in the cytoplasm, and subsequent translation of the transgene (74). The suppression in either of the abovementioned steps may have resulted in lack of SFV-encoded transgene expression in macrophages as we observed. Furthermore, fresh isolated peritoneal mouse macrophages were also resistant to infection with the replication-deficient SFV-DsRed virus as was confirmed by absence of fluorescent signal in the infected cells (data not shown). In contrast to our results, a study from 1976 described that a replication-competent SFV was able to infect and multiply at very low yields within peritoneal mouse macrophage cultures *in vitro* (75). The authors tested the presence of SFV within cell growth medium of peritoneal macrophages post-infection by titrating plaque forming units in chick embryo cell cultures. Similarly, another study from 1988 reported low susceptibility of mouse spleen-derived Mac-1-positive cells to infection with replication-competent or first-generation avirulent SFV (76). The differences between the protocols and SFVs used in our study and studies by van der Groen et al. or Wu et al. make it difficult to compare the results. However, a possible explanation for the macrophage low susceptibility toward SFV in the studies mentioned (75, 76) could be that the *ex vivo* isolated peritoneal or spleen cells, which the authors used as a source of macrophages, also contained other cell types that served as host cells for SFV multiplication. The main difference though is that the authors used replication-competent SFVs, including a wt SFV, which was isolated from mosquitos and was produced by replication in chick embryo cell cultures, or first-generation avirulent SFV. Our data based on use of pure macrophage cultures clearly show that mouse and human macrophages are resistant to infection with SFV particles that are based on the replication-deficient rSFV1 vector and that have been produced in BHK-21 cells.

Infection with SFV inhibited proliferation of BHK-21 and LLC cells *in vitro* independent on whether SFV-encoded TNF-α or IFN-γ. The inhibition of cancer cell growth is likely caused by the ability of SFV particles to induce p53-independent apoptosis in infected cells, which is linked to viral RNA replication and appearance of double-stranded RNA intermediates in cell cytoplasm (21). This property is of particular interest for lung cancer therapy since a mutated p53 is one of the key molecular markers in many solid cancers, including human lung cancers (77). In fact, the target LLC cancer cells used in this study were shown to undergo cell death after infection with replication-deficient rSFV particles. The infected cancer cells were shown to express rSFV-encoding transgene while undergoing cell death. Thus, rSFV has therapeutic potential not only as a transgene delivery vehicle but may also elicit a direct oncolytic effect on cancer cells. Moreover, intratumoral injection of rSFV particles has been shown to inhibit tumor growth in preclinical mice models by induction of apoptosis (78).

Early after infection with rSFV particles the host cells shut down the translation of endogenous cellular proteins (79), whereas the heterologous protein encoded by rSFV is produced at high levels (80). In the present study, we embraced this property in order to produce tumor-suppressive cytokines within cancer cells. We showed that LLC cells can produce and release rSFV-encoded TNF-α and IFN-γ in the cell culture medium at levels that are functional *in vitro*. Whereas vdIFN-γ was present at 100 ng levels in the supernatant, the amount of vdTNF-α was in several nanograms. The relatively low levels of vdTNF-α that we detected might be explained by the lack of translational enhancer in a vector, as well as by the fact that TNF-α is primarily produced as a transmembrane protein (81), and cleavage of the transmembrane form is needed before soluble TNF-α can be released into the cell culture medium (82).

Nevertheless, the levels of vdTNF-α detected were sufficient to elicit a functional response *in vitro* as shown by induction of cell death in mouse fibroblast cell line L929. TNF-α is known to induce either caspase-dependent apoptotic cell death or caspase-independent programmed necrotic cell death (necroptosis) downstream of TNFR1 signalling depending on microenvironment and cell type (83, 84). We induced mainly necrotic cell death in L929 cells in a dose-dependent manner after treatment with either vdTNF-α or rTNF-α, which is in agreement with previous reports showing that L929 cells undergo caspase-independent necroptosis in response to TNF-α treatment (68, 85). In accordance with a previous study (65) we also observed an annexin V-positive/PI-negative cell population after treatment with vdTNF-α, which may represent not only apoptotic but also primary necrotic cells according to studies showing phosphatidylserine exposure during early necrosis (oncosis) before the integrity of the membrane is lost (86, 87). By inducing fibroblast cell death *in vitro*, we verified that SFV-encoded TNF-α is biologically active. However, *in vivo* effects of TNF-α differ and comprise increased vessel permeability used to improve drug penetration (43–45) and destruction of tumor vasculature (41, 42) leading to hemorrhagic tumor necrosis (40, 88). By using recombinant cytokines, we also showed that TNF-α is a cytokine that may induce cytotoxicity in mouse lung carcinoma cells but only when combined with IFN-γ. Such observations can be explained by a previously reported IFN-γ property to enhance TNF-α receptor expression (51, 52), thus sensitizing cancer cells to TNF-α-induced cell death. Furthermore, in the context of cancer immunotherapy TNF-α may serve as an adjuvant in order to enhance T-cell infiltration (46, 89).

In this study, we showed that rSFV-encoded IFN-γ was functional in activating macrophages *in vitro* toward a tumoricidal phenotype, which is consistent with previous studies showing that IFN-γ renders macrophages tumoricidal *in vitro* (34–36). We observed that in order to activate macrophages, presence of both vdIFN-γ and the TLR2/1 agonist Pam3 was required. Also previous studies showed that two signals, IFN-γ in combination with a second signal are required to render macrophages tumoricidal (39) and able to induce NO production (90). It is known that classically activated or M1 macrophages increase the expression of inducible NO synthase and, thus, produce NO (91). We showed that vdIFN-γ synergized with TLR2/1 agonist Pam3 to induce NO production in macrophages in a concentration-dependent manner, similarly as observed by combining rIFN-γ with another TLR agonist, CpG-containing DNA, respectively, in a previous study (90). Our observation that high level of NO, which was produced by activated macrophages, correlated with increased cancer cell growth inhibition is in agreement with the early studies reporting that tumoricidial abilities of macrophages *in vitro* are based on NO being cytotoxic to cancer cells (92, 93).

Macrophages are common immune cells in many tumors and often are considered to inhere a pro-tumoral phenotype associated with angiogenesis, metastasis, and suppression of T cell activation (94). By contrast, M1 macrophages suppress tumor growth and angiogenesis (95) and might be positively associated with patient survival (95–97). Several *in vivo* studies have shown the therapeutic benefit of redirecting tumor-associated macrophages to M1 phenotype by agents such as TLR9 ligand CpG in combination with antibody to IL-10R (88), TLR7/8 agonist 3M-052 (98), or liposomal nanoparticle-encapsulated hydrazinocurcumin (99). Previous studies suggest IFN-γ as a good candidate for tumor-associated macrophage re-education due to the ability of IFN-γ to re-polarize tumor-associated macrophages toward an M1 phenotype *in vitro* (100) and its ability to render macrophages tumor-suppressive in mouse tumor models *in vivo* (31, 37–39). The results from this study suggest that SFV-delivered vdIFN-γ is as efficient as rIFN-γ in rendering macrophages tumoricidal *in vitro*, which along with our finding that macrophages but not cancer cells are resistant to SFV infection encourages a possible application of SFV-Ifng vector for activation of tumor-associated macrophages toward tumor-suppressive M1 phenotype in further studies *in vivo*. Moreover, either IFN-γ or TNF-α delivery by rSFV vector may comprise not only a direct effect of the rSFV-delivered cytokines but may also benefit from the inherent ability of SFV vector to induce p53-independent apoptosis (21) and elicit endogenous type-I IFN responses, which may be required for the therapeutic effect of a vector-encoded cytokine (3).

In summary, the present study shows that rSFV can efficiently infect lung carcinoma cells *in vitro* and exploit them for production of functional rSFV-encoded TNF-α and IFN-γ. The rSFV-encoded IFN-γ was able to activate macrophages toward an antitumor phenotype *in vitro*. Our results set the basis for application of SFV-Tnfa and SFV-Ifng vectors to modulate the cytokine milieu in the tumor microenvironment in further studies using mouse models for lung cancer.

## Ethics Statement

This study was carried out in accordance with the recommendations of The Norwegian Regulation on Animal Experimentation, Norwegian Food and Safety Authority.

## Author Contributions

AZ and AC conceived the study. All authors contributed to the design of experiments, data analysis, and interpretation of data. BK, EM, PC, IJ, BS, and IØ performed experiments. BK wrote the manuscript; and IØ, AC, and AZ contributed in writing the manuscript. All authors critically revised the manuscript and approved the final version. AC and AZ contributed equally to this work.

## Conflict of Interest Statement

The authors declare that the research was conducted in the absence of any commercial or financial relationships that could be construed as a potential conflict of interest. The reviewer CS and handling Editor declared their shared affiliation.

## Abbreviations

3H-TdR, [methyl-3H]-thymidine; BHK-21, baby hamster kidney fibroblasts; BMDM, bone marrow-derived macrophage; CM, conditioned medium; cpm, counts per minute; DsRed, *Discosoma* sp. red fluorescent protein; FBS, fetal bovine serum; FITC, fluorescein isothiocyanate; GFP, green fluorescent protein; HMDM, human monocyte-derived macrophage; IFU, infectious units; LLC, Lewis lung carcinoma; MOI, multiplicity of infection; NO, nitric oxide; NO2-, nitrite; Pam3, Pam3CSK4; PBS−/−, phosphate-buffered saline without magnesium and calcium; PBS+/+, phosphate-buffered saline with magnesium and calcium; PI, propidium iodide; rIFN-γ, recombinant interferon-γ; rSFV, recombinant Semliki Forest virus; rTNF-α, recombinant tumor necrosis factor-α; SeV, Sendai virus; SFV, Semliki Forest virus; TLR, toll-like receptor; vdIFN-γ, vector-derived interferon-γ; vdTNF-α, vector-derived tumor necrosis factor-α.
